# Harnessing *Terminalia arjuna* in Next Generation Phytomedicine: Cutting Edge Nutraceutical Trends Shaping Food and Health

**DOI:** 10.1002/fsn3.72352

**Published:** 2026-09-29

**Authors:** Ishani Banerjee, Fahika Nazeerulla, Manash Kumar Paul, Sujit Kumar Bhutia, Deblina Basu, Subhadip Mukhopadhyay

**Affiliations:** ^1^ Department of Biotechnology Manipal School of Life Sciences, Manipal Academy of Higher Education Manipal India; ^2^ Department of Radiation Biology and Toxicology Manipal School of Life Sciences, Manipal Academy of Higher Education Manipal India; ^3^ Department of Life Science National Institute of Technology Rourkela Rourkela India; ^4^ Department of Microbiology New York University (NYU) Langone Health New York USA

**Keywords:** arjunolic acid, cancer, cardioprotectant, nutraceuticals, phytomedicines, *Terminalia arjuna*

## Abstract

Phytomedicines are increasingly favored as alternatives to modern treatment due to their enhanced efficacy and cultural relevance in preserving traditional healing knowledge while supporting sustainable healthcare practices. Among them, *Terminalia arjuna* stands out as a widely consumed dietary and Ayurvedic remedy. This review explores the pharmacological evidence for *T. arjuna* and its relevance to food science and toxicology, unveiling its molecular mechanisms in health and disease. They target the axis of p53‐PARP mediated apoptosis, endoplasmic reticulum stress mechanisms, mitochondrial integrity, and TGF‐β mediated remodeling processes. *T. arjuna*'s major bioactive constituents like arjunic acid, arjunolic acid, and casuarinin, to name a few, possess antioxidant, anti‐inflammatory, cardioprotective, renal, and liver‐protective capacities. In this work, we critically evaluate the strength of preclinical versus clinical evidence and identify the gaps in bioavailability, safety, and herb‐drug interaction data, beyond its pharmacological profile, to establish *T. arjuna*'s translational potential. This study further explores *T. arjuna* as a candidate for modern nutraceutical and functional food development while highlighting the need for future validations before its full potential is confirmed.

## Graphical abstract

This graphical abstract illustrates the complete translational pipeline of *Terminalia arjuna* in modern phytomedicine and functional food development. It is rich in key bioactive compounds like arjunolic acid, arjungenin, flavonoids, and tannins which serve as the foundation for the plant's versatile medicinal uses and offers broad therapeutic benefits against heart disease, cancer, infections, brain disorders, and liver injury, as well as slow‐healing wounds. To translate these health benefits into shelf‐stable foods and supplements, researchers rely on physicochemical profiling, formulation testing, stability checks, and animal studies, highlighting the formulation of next‐generation nutraceuticals. Alongside product development, systematic safety evaluations cover potential herb‐drug interactions, preclinical toxicity screenings, and human clinical trials to ensure that *T. arjuna*‐based nutraceuticals are both effective and safe for long‐term use.

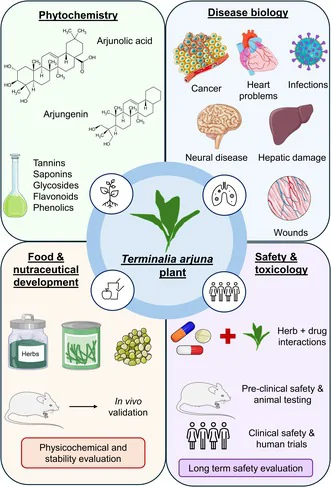

## 1 Introduction

Across different civilizations, indigenous healing practices known as “Traditional medicine” encompass Chinese herbal medicine, Indian Ayurveda, Unani medicine (Heirangkhongjam and Ngaseppam 2018), along with other regional practices of Africa, Europea, American Aborigines, Indonesian Jamu, and Australian bush medicines (Patwardhan et al. 2023). Herbal medicines continue to serve as a primary source of healthcare for about 80% of the world's population (Latif and Nawaz 2026). This growing recognition was also reflected at the 78th World Health Assembly held in May 2025, where the World Health Organization (WHO) launched the Global Traditional Medicine Strategy 2025–2034 to strengthen evidence‐based traditional, complementary, and integrative medicine practices worldwide (Burki 2025). In low and middle‐income regions, limited access and high cost of conventional medicine have driven the increasing use of phytomedicines, whereas in higher‐income countries, their popularity is fuelled by growing consumer demand for natural and holistic alternatives (Bareetseng 2022; Møller et al. 2024).

Phytomedicines being plant‐derived medicines are widely used in the prevention and management of various diseases. Traditionally incorporated into the diet through crude formulations like botanical extracts, polyherbal mixtures, and essential oils, which are explored as therapeutic or nutraceutical agents (Dwivedi and Chopra 2014). Plants used in traditional medicine have also contributed to modern cancer treatment. For example, vinca alkaloids, vincristine, vinblastine, and vinorelbine derived from *Catharanthus roseus* were used to treat various hematological malignancies and solid tumors (Martino et al. 2018). This review focuses on the medicinal plant *Terminalia arjuna*, popularly known as Arjuna, which belongs to the family of Combretaceae. It is an evergreen tree attaining a height of about 20–30 m and is found throughout South Asia, mainly in India, Sri Lanka, Burma, and Mauritius (Kapoor et al. 2014). *T. arjuna* can be categorized as a dietary phytomedicine, rich in tannins, alkaloids, glycosides, flavonoids, triterpenoids, polyphenols, phytosterols (proanthocyanidins, β‐sitosterol), and some minerals (like Cu, Zn, Mg, Ca) (Bhattacharjee et al. 2019). These constituents have contributed to its extensive use in the management of diverse diseases exhibiting activities like anti‐tumoral (Elsherbiny and Al‐Gayyar 2016), anti‐inflammatory (Vijayalakshmi et al. 2023), anti‐cancer (Manna et al. 2020), anti‐oxidant (Verma and Vinayak 2009), anti‐microbial (Jaiswal and Kumar 2016), hypolipidaemic (Sawale et al. 2016) wound healing (Chaudhari and Mengi 2006), cardioprotective (Mohanty et al. 2019), and anti‐diabetic (Aamir et al. 2022) properties. Despite the wide range of pharmacological activities, their effectiveness has not yet been translated into standardized, clinically validated nutraceutical products. This review brings together the current evidence on the phytochemistry and pharmacology of *T. arjuna*, with an emphasis on its use in nutraceutical and functional food development (Tables 1, 2, 3).

**TABLE 1:** Current evidence for *Terminalia arjuna* across major therapeutic areas, categorized as in vitro, animal/in vivo, or clinical evidence.

Therapeutic area	In vitro	Animal/in vivo	Clinical
Cancer	Broad cell‐line studies on TAE, arjunic acid, casuarinin, nanoparticles	Ehrlich ascites, DLA, DMBA hamster models	No human cancer trials identified
Cardiovascular	Cardiomyocyte and platelet studies	CoCl_2_, doxorubicin, sodium nitrite, isoproterenol, atherosclerosis, fibrosis models	Many human trials on *Terminalia arjuna* formulations are reported
Hepatic	Hepatocyte and liver‐cell oxidative stress studies	APAP, cadmium, arsenic, fluoride, CCl_4_ models	No human liver trials identified
Renal	Perfused kidney/renal cell oxidative stress studies	Cisplatin, APAP, fluoride, stone‐forming and renal injury models	No human renal trials identified
Multiorgan oxidative stress	DPPH and antioxidant assays supporting extract activity	CCl_4_, NaF, lead acetate models	No human studies identified
Wound healing and dermatoprotective effects	Antimicrobial activity against wound associated microorganisms	Rat wound models	Evaluation of TEWL, microcirculation, skin density, and skin thickness
Infectious diseases	Antibacterial and antifungal assays; HSV‐2 cell assays	Not clearly established	No clinical studies identified
Neuroprotective	Neuronal/cell‐based protection against oxidative damage	Cerebral ischemia, hypobaric hypoxia, anxiety models in rodents	No human neurological trials identified
Antidiabetic	Enzyme inhibition, insulin‐signaling, docking, cellular antioxidant studies	STZ, alloxan, diabetic complication models	Comparative trial (*n* = 60) TAE + metformin vs. sitagliptin + metformin for 3 months

## 2 Prevalence in Ayurveda and Indian Traditional Medicine

One of the oldest and most ancient systems of medicine in India is “Ayurveda” which aims to integrate the balance of the body, mind and spirit (Sowmiya et al. 2025). The U.S. National Health Interview Survey 2007 Complementary and Alternative Medicine (CAM) Supplement revealed that 214,000 adults used an Ayurvedic product, which was a 28% increase from the 2002 survey on CAM use (Barnes et al. 2008). Among the many medicinal plants used in Ayurveda, the bark of *T. arjuna* stands out for its role in supporting cardiovascular health, with references in ancient Indian medicinal literature texts such as the *Charaka Samhita* and *Astang Hridayam* (Rajaram et al. 2024). Traditionally, it is known as “hritshool”, its stem bark powder acts as a cardiac stimulant for angina, heart failure, and atherosclerosis, etc. (Maulik and Talwar 2012). Although Arjuna has a long history of use in traditional medicine, its documented therapeutic use is largely concentrated in India, particularly within Ayurvedic practice. Pharmaceutical companies produce numerous patented formulations featuring it as a key ingredient in *Arjunarishta*, *Arjun‐ghrita*, *Arvindasava*, *Arjun ksheer‐pak*, and *Lakshadi guggulu*.

*T. arjuna* bark powder has also traditionally been administered in the form of ghee (clarified butter), as an alcoholic decoction, or mixed into boiled milk (Gaikwad and Jadhav 2018). *Arjunarishta*, a traditional hydroalcoholic Ayurvedic formulation made from *T. arjuna, Madhuca indica*, *Vitis vinifera* , and *Woodfordia fruticose* (Mehta 2020), has long been used to regulate blood pressure and cholesterol, strengthen heart muscles, enhance overall cardiac performance, alongside exerting anti‐diabetic effects (Shengule et al. 2018). Treatment with *Arjunarishta* unveiled the anti‐glycemic, anti‐hyperlipidemic, and anti‐hypertriglyceridemic potential of the formulation, mediated by decreased tumor necrosis factor‐α (TNF‐α), increased peroxisome proliferator activated receptor gamma coactivator‐1α (PGC‐1α), and insulin receptor substrate‐1 (IRS‐1), thereby improving insulin sensitivity (Shengule et al. 2018). As described in *Chakradatta*, *Arjuna Ksheera Paka* is a cardiotonic prepared from dry tree bark powder, cow milk, and water—with its name derived from Sanskrit words “*ksheeram*” (milk) and “*pakam*” (to boil). It shows strong anti‐inflammatory effects in vivo, but comparatively lower antioxidant activity in vitro (Dube et al. 2017).

## 3 Phytochemistry and Compound Characterization

*T. arjuna* bark contains four major phytochemical classes: triterpenoids (arjunic acid, arjunolic acid (AA), arjunetin, arjungenin, arjunglucosides I and II), tannins (15 varieties, including casuarinin and punicalagin), flavonoids (luteolin, quercetin, kaempferol, baicalein), and phenolic acids (gallic acid, ellagic acid) which collectively form the basis of its traditional use as a cardiotonic (Alam et al. 2022; Chaudhari and Mengi 2006; Jain et al. 2009). Its individual components show different activity like arjunic acid and AA are cytotoxic and antioxidant (Mythili et al. 2012; Saxena et al. 2007), luteolin and gallic acid/ethyl gallate inhibit several cancer cell lines (Pettit et al. 1996; Ryu et al. 1994), and triterpenoids from its bark extract show antifungal/antibacterial activity (Jain et al. 2009). Tannins are responsible for astringent, anticancer, antibacterial and wound healing properties (Dwivedi 2007). These phytoconstituents are not distributed evenly across the plant. Comparative screening of leaf, bark, and fruit extracts across a panel of solvents showed that flavonoids, tannins, and phenolics are detectable in all three tissues (Singh et al. 2018). The stem bark is the main source of arjunic acid, arjunolic acid, arjungenin, and arjunglucosides I/II while the root bark yields a distinct pair of glycosides, arjunoside III and IV (Anjaneyulu and Rama Prasad 1982). Quantitatively, HPLC‐based sampling of stem bark across different geographical locations in India found substantial site‐to‐site variation in arjungenin and arjunic acid content and their concentrations also varied depending on geographic source (Kalola and Rajani 2006). Different extraction methods used for *T. arjuna* affect not only yield but also the composition of the resulting extract. Alcoholic solvents such as isopropanol and ethanol efficiently extracted arjunic acid, whereas hexane did not, while extracts obtained with solvents of different polarity showed only nine phytochemicals in common out of 30 identified compounds (Saha et al. 2012). Similarly, ultrasound‐assisted extraction produced higher phenolic and flavonoid contents than conventional extraction, partly due to reduced degradation during prolonged heating (Tahir, Akhtar, Saeed, et al. 2025). Therefore, extraction method can influence both composition and biological activity of *T. arjuna* preparations.

## 4 Role of Secondary Metabolites Derived From *T. arjuna* in Disease Regulation

### 4.1 Anticancer Effects

TAE and its major bioactives show antiproliferative activity across a wide range of cancer cell lines including MCF‐7 breast cancer, A‐549 non‐small‐cell lung cancer, Jurkat leukemia, U87 glioma, and RMI‐7951 melanoma cells and induce apoptosis in HL‐60 and HepG2 cells (Greco et al. 2021; Kuo, Hsu, Lin, Chang, and Lin 2005; Kuo, Hsu, Lin, Lin, et al. 2005; Sivalokanathan et al. 2006). Nearly all its compounds converge on mitochondrial, ROS‐, or caspase‐mediated apoptosis as their core mechanism, despite their differences (Figure 1). However, all evidence supporting *T. arjuna's* anticancer activity is derived from cell‐culture and animal studies, with no human clinical trial evaluating *T. arjuna* for cancer.

**FIGURE 1:**
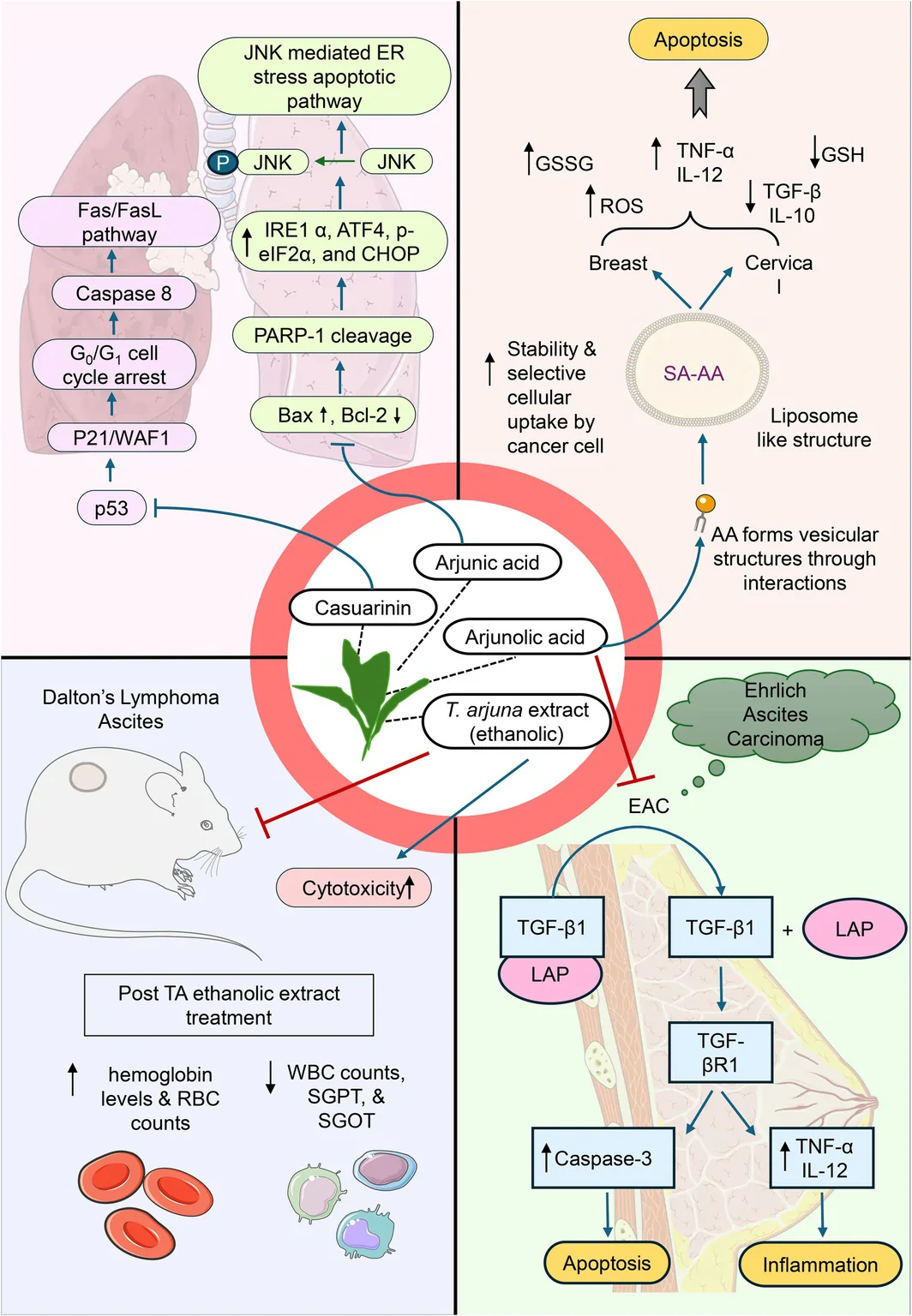
Molecular and cellular mechanisms underlying the anti‐cancer potential of *Terminalia arjuna*. The schematic diagram illustrates the proposed pathways which show how the extract and major bioactives of *T. arjuna*—casuarinin, arjunic acid and AA exert anticancer effects. Casuarinin induces apoptosis through activation of the p53/Fas–FasL signaling, G_0_/G_1_ cell cycle arrest, caspase‐8 activation, PARP‐1 cleavage, and upregulation of Bax with concomitant downregulation of Bcl‐2 in lung cancers. This compound also triggers the JNK dependent ER stress pathway via IRE1α, ATF4 and CHOP signaling, leading to apoptosis. AA forms liposome‐like vesicular structures enhancing selective uptake by cancer cells and promoting ROS generation with TNF‐α and IL‐10 upregulation. In DLA, TAE elevates hemoglobin and RBC levels, normalizes hepatic enzyme profiles, and enhances cytotoxicity while in EAC, AA lowers TGF‐β1/LAP, along with downregulating TNF‐α and increasing IL‐10 and caspase‐3, which collectively mediate apoptosis and inflammation.

#### 4.1.1 Apoptosis and Cell Cycle Arrest

In A549 and H460 non‐small‐cell lung cancer cells, arjunic acid triggers classic apoptotic hallmarks like apoptotic body formation, cell shrinkage, and PARP cleavage (Chaitanya et al. 2010) while attenuating expression of anti‐apoptotic Bcl‐2, and upregulation pro‐apoptotic Bax (Figure 1). It activates ER stress via IRE1α, CHOP, ATF4, p‐eIF2α, an effect dependent on JNK phosphorylation, triggering death and enhancing the sub‐G1 population. JNK inhibition with SP600125 blocks IRE1α/CHOP induction and cytotoxicity, confirming JNK's pivotal role (Joo et al. 2016). In HepG2 liver cancer cells TAE upregulates p53, the “guardian of the genome” which orchestrates cell cycle arrest, DNA repair and activates caspase‐3 confirming caspase‐mediated apoptosis (Sivalokanathan et al. 2006). These findings suggest that arjunic acid may act through several overlapping pathways to trigger apoptosis, though more studies using different are needed to confirm this. A second compound, casuarinin, a hydrolysable tannin isolated from the bark (Tahir, Akhtar, Bishoyi, et al. 2025) illustrates the same convergence through a different route. Casuarinin induced dose‐dependent G_0_/G_1_ cell‐cycle arrest in A549 and MCF‐7 cancer cells. In A549 cells, it is mediated by p53 upregulation and p21/WAF1 induction, pointing to a p53‐dependent mechanism that inhibits cyclin–CDK complexes while in MCF7 cells casuarinin elevates p21/WAF1 without increasing p53, indicating that p21 upregulation alone can enforce G_0_/G_1_ arrest‐. This demonstrates that the same compound can reach a similar endpoint through different molecular paths depending on cell type. Casuarinin additionally engages the extrinsic apoptotic pathway via Fas/FasL and caspase‐8 activation (Kuo, Hsu, Lin, Chang, and Lin 2005; Kuo, Hsu, Lin, Lin, et al. 2005) and separately shows antiviral activity against herpes simplex virus Type 2 (HSV‐2) (Yang et al. 2000) and cytotoxicity in Jurkat cells (Greco et al. 2021), extending its relevance beyond oncology alone.

#### 4.1.2 Animal Model Evidence

Moving beyond cell culture, arjunolic acid (AA) has been tested in an Ehrlich Ascites Carcinoma (EAC) mouse model—a step up in evidence quality, as it captures tumor growth in a living system rather than isolated cells. AA reduces tumor growth by multiple mechanisms: lowering oxidative stress and malondialdehyde (MDA) levels, abrogating TGF‐β1 signaling by downregulating TGF‐β1, latency‐associated peptide (LAP), and TGF‐βR1/ALK‐5, restoring immune balance (decreased TNF‐α, increased IL‐10), and activating caspase‐3 (Elsherbiny and Al‐Gayyar 2016). Similarly, ethanolic TAE prolonged survival in Dalton's Lymphoma Ascites (DLA) mice (by 60%–87.5% depending on dose) alongside improving hematological and liver enzyme parameters (Figure 1). ETA also suppresses solid tumor growth, reducing tumor volume and extending lifespan by about 35% (Ganesan et al. 2010). Unlike the EAC and DLA models above, which treat already established tumors, the 7,12‐Dimethylbenz[a]anthracene (DMBA) hamster oral model tests chemoprevention. In this model, oral administration of TAE (500 mg/kg) reduced tumor incidence, burden, and volume, normalized antioxidant status, and lowered lipid peroxidation (Table 2), highlighting its chemopreventive antioxidant potential in early‐stage oncology‐adjacent nutraceutical strategies (Dhanarasu et al. 2010). Despite differing tumor types, compounds, and treatments across these three animal models, a similar antioxidant‐mediated, immune‐modulating pattern recurs, reinforcing the cell‐culture findings discussed earlier and building a compelling case for translation to human trials.

**TABLE 2:** Bioactive metabolites of *Terminalia arjuna* and its biological targets.

Metabolite/extract	Organ/system	Study type	Disease/experimental model	Targets/effects of TA	References
Arjunic acid	Lung (NSCLC)	In vitro	A549 and H460 cells	Leads to PARP cleavage; increased sub‐G1 phase population; IRE1 α, ATF4, p‐eIF2α, and CHOP upregulation, Bax upregulation; Bcl‐2 downregulation; activates JNK mediated ER stress apoptotic pathway	Joo et al. (2016)
Arjunic acid	Human oral, ovarian, liver cancer	In vitro	KB, PA1, Hep‐G2, WRL‐68	Cytotoxic activity; anti‐oxidant activity; anti‐tumor activity; anti‐inflammatory activity	Saxena et al. (2007)
Casuarinin	Lung (NSCLC)	In vitro	A549 cells	Upregulation of p53; induction of p21/WAF1 protein; G0/G1 cell cycle arrest; caspase‐8 activation; activation of Fas/FasL pathway	Kuo, Hsu, Lin, Chang, and Lin (2005)
Casuarinin	Breast cancer	In vitro	MCF‐7 cells	No significant changes on the expression of p53; Induction of p21/WAF1 protein; G0/G1 cell cycle arrest; caspase‐8 activation; Activation of Fas/FasL pathway	Kuo, Hsu, Lin, Lin, et al. (2005)
Arjunolic acid	Mammary adenocarcinoma	In vivo	Ehrlich ascites carcinoma (murine model)	Upregulation of TGF‐β1, LAP, TGF‐R1; reduced MDA; elevated TNF‐α, IL‐12, caspase‐3 and IL‐10	Elsherbiny and Al‐Gayyar (2016)
Arjunolic acid	Heart	In vivo	Doxorubicin induced cardiotoxicity in albino Wistar rat cardiomyocytes	Attenuation of p53 and p38 activation; Preserves Δψm; Prevents cytochrome c release; Inhibits mitochondrial translocation of Bax; upregulated Bcl‐2 and Bcl‐xL; inhibits caspase‐3 activation and cleavage of PARP; Inhibits activation of JNK MAPK; prevents mitochondrial mediated apoptosis	Ghosh et al. (2011)
Arjunolic acid	Heart	In vivo	ISO‐induced myocardial necrosis in Wistar rats	Elevated antioxidant levels of SOD, CAT, GPx, GST, GSH; reduces MPO levels; anti‐platelet effect; anti‐coagulant effect; hypocholesterolemic effects	Sumitra et al. (2001)
Arjunolic acid	Heart	In vivo	Sodium nitrite‐induced toxicity on cardiac tissue in Sprague Dawley rats	Increased glutathione and mitochondrial cytochrome C oxidase; TNF‐α, IL‐1β suppression; elevated IL‐4, IL‐10; blocked JNK activation and activation of caspase‐3/8/9; Prevents both intrinsic and extrinsic apoptosis	Al‐Gayyar et al. (2014)
Arjunolic acid	Heart	In vivo	Anti‐fibrotic role of AA in hypertrophy‐associated cardiac fibrosis in Wistar rats	Upregulation of PPARδ; Downregulation of TAK1, collagen‐1 and collagen‐3 expression, ECM proteins; blocks non‐canonical TGF‐β signaling pathways (NF‐κB, p65, p38 MAPK); regresses cardiac fibrosis and hypertrophy; improves myocardial function during hypertrophy	Bansal et al. (2017)
Arjunolic acid	Liver	In vivo	Acetaminophen‐induced acute hepatotoxicity in albino Wistar rats	Downregulation of CYP2E1 activity (metabolic inhibition; less NAPQI formed), oxidative/nitrosative stress mediators (ROS, NO, H_2_O_2_, oxidative damage); upregulated antioxidant defenses (GSH, redox balance); blocks JNK activation; inactivation of Bcl‐2 and Bcl‐xL; protection from mitochondrial permeabilization and cell death	Ghosh et al. (2010b)
Arjunolic acid	Liver	In vivo	Arsenic (NaAsO_2_) induced cytotoxicity in hepatocytes of Swiss albino mice	Downregulation of ALT, ALP level; enhances cellular antioxidants SOD, CAT, GPx, GR, GST levels; reduces GSSG and restores GSH/GSSG balance	Manna, Sinha, Pal, and Sil (2007)
Arjunolic acid	Liver	In vitro	Sodium fluoride (NaF) induced cytotoxicity in Swiss albino mice hepatocytes	Downregulation of ALT & ALP, ROS, MDA, protein carbonylation; Increased antioxidant enzyme activities (SOD, CAT, GST, GR, GPx) and GSH & total thiols; Enhanced cell viability; Attenuated necrosis	Ghosh et al. (2008)
Arjunolic acid	Liver	In vivo	Cadmium‐induced hepatic dysfunction in Swiss albino mice	Enhances cellular antioxidants SOD, CAT, GPx, GR, GST levels; downregulated ALT, ALP, LDH, MDA level, lipid peroxidation levels; Downregulation of Bad; Upregulated Bcl‐2; preserves Δψm; prevents activation of caspase‐9, caspase‐3, and PARP cleavage; decreases Fas, caspase‐8, and t‐Bid levels	Pal et al. (2011)
Arjunolic acid	Kidney	In vivo	Cisplatin induced nephrotoxicity in Sprague Dawley rats	Downregulation of NADPH oxidase, MDA, NF‐κB, TNF‐α, IL‐1β, TGF‐β, TGFβR1, caspase‐3/8/9; upregulated SOD activity and overall antioxidant defense; prevents lipid peroxidation, inflammation, fibrosis, and apoptosis	Elsherbiny et al. (2016)
Arjunolic acid	Kidney	In vivo	Cisplatin‐induced nephrotoxicity in Sprague Dawley rats	Attenuation of NF‐kB activity, TGF‐b expression, Kim‐1; upregulation of Bcl‐2 proteins	Sherif (2015)
Arjunolic acid	Kidney	In vivo	Acetaminophen‐induced acute renal toxicity in albino rats of Wistar strain	Downregulation of ROS, lipid peroxidation, protein oxidation, TNF‐α, iNOS, NO, caspase‐9, caspase‐3; Upregulates SOD, CAT, GST, GR, GPx, GSH, total thiols, FRAP; radical scavenging (ROS + NO) activity; maintains redox balance; Prevents peroxynitrite formation	Ghosh et al. (2010a)
Arjunolic acid	Testes	In vivo	Arsenic (NaAsO_2_) induced testicular oxidative stress in Swiss albino mice	Upregulates/restores SOD, CAT, GPx, GR, GST, total thiols; maintenance of structural and functional integrity of seminiferous tubules (protection of testicular tissue); Downregulated GSSG levels, ROS and RNS levels (superoxide, hydroxyl radical, peroxyl radical, NO, H_2_O_2_); Prevents lipid peroxidation, protein carbonylation	Manna et al. (2008)
Arjunolic acid	Brain	In vivo	Cerebral ischemia–reperfusion (stroke model)	Enhanced SOD, CAT, GPx, GR; attenuated MDA, nitrite, mitochondrial ROS; increase in Na+/K + ‐ATPase activity	Yaidikar and Thakur (2015)
Arjunolic acid	Liver	In vitro	Hepatocellular carcinoma (HepG2)	Upregulation of p53; caspase‐3‐mediated apoptosis	PMID: 16534840
Arjunolic acid	Liver and kidney	In vivo	NaF induced hepatic and renal oxidative damage in Swiss albino mice	Normalized antioxidant enzymes (SOD, CAT, GST, and GSH) & thiols; prevented oxidative damage in liver and kidney; showed free radical scavenging activity comparable to Vitamin C	Sinha et al. (2007)
Alcoholic *Terminalia arjuna* bark extract (alcoholic TAE)	Liver, kidney and heart	In vivo	CCl_4_‐induced oxidative stress; hepatotoxicity; renal oxidative injury	Restored SOD, CAT, GST and GSH; downregulated GPT, ALP and lipid peroxidation; prevents oxidative stress; modulates CYP450 to reduce CCl_4_ bioactivation; protects hepatocytes & renal tissue	Manna et al. (2006), Manna, Sinha, and Sil (2007)
Alcoholic TAE	Liver and kidney	In vivo	Lead acetate‐induced oxidative stress; hepatotoxicity; nephrotoxicity	Restored SOD, CAT, GR, GPx, GST and GSH; downregulated ALT, AST, ALP and lipid peroxidation; decreases serum urea, uric acid, bilirubin and creatinine levels; prevents oxidative stress; restores hepatic, renal, and neural function	Mohammed Raouf et al. (2013)
Alcoholic TAE	Heart	In vivo	Doxorubicin‐induced cardiotoxicity	Restored SOD, CAT and GSH; downregulated ROS, RNS and lipid peroxidation; reducing cell injury and cell death	Singh et al. (2008)
Alcoholic TAE	Kidney (isolated perfused rat kidney)	Ex vivo	Oxidative stress–induced renal injury	Increases GSH and GPx levels; reduced GOT and GPT levels; protects against ROS‐mediated oxidative damage	Raj et al. (2012)
Alcoholic TAE	Heart	In vivo	ISO‐induced cardiac stress in Wistar rats	Elevated antioxidant levels of SOD, CAT, GPx, GR, GSH; free radicle scavenging; decreased lipid peroxidation, NO synthesis; prevented JNK1/2 and c‐jun phosphorylation; inhibited caspase 3; Bcl‐2 upregulation; Bax downregulation	Thangaraju et al. (2020)
Alcoholic TAE	T‐cell lymphoma	In vivo	Dalton's Lymphoma Ascites (DLA) in mice	Increased hemoglobin and RBC; reduced WBC, SGPT, SGOT, tumor volume and tumor growth; strong cytotoxic effect	Ganesan et al. (2010)
Alcoholic TAE	Oral cancer	In vivo	DMBA‐induced oral cancer (hamsters)	Upregulated ascorbic acid, vitamin E, GSH, SOD, CAT, GPx (antioxidants); downregulated lipid peroxidation	Dhanarasu et al. (2010)
Alcoholic TAE	Brain	In vivo	Cerebral ischemia (rat stroke)	Decrease in infarct volume, neurological deficits, vascular leakage; Restores antioxidant & ROS‐scavenging activity; reduced MMP‐2 & MMP‐9, COX‐2, TIMP‐1/2 and; preserves tight junction proteins (Occludin, Claudin‐5, ZO‐1); decreased neuronal degeneration and BBB leakage	Kaliyappan et al. (2021)
*Terminalia arjuna* aqueous extract (TAE)	Heart	In vivo	Atherosclerosis in ApoE −/− mice	Upregulated IL‐18/IL‐18R, NF‐κB levels; downregulated CD36, MMP‐9, VCAM‐1, ICAM‐1; elevated PPAR‐γ, LXR‐α levels; Improved lipid profiles	Bhat et al. (2018)
TAE	Kidney (MDCK cells)	In vitro	Oxalate‐induced renal epithelial injury	Decreased CaOx–cell adhesion; reduced oxidative stress, caspase‐3 activation, apoptosis; increased cell viability	Mittal et al. (2017), Mittal et al. (2016a)
TAE	Kidney (MDCK cells)	In vitro	Oxalate‐induced renal epithelial injury	Reduced nuclear condensation, nuclear fragmentation, ROS production, apoptosis; stabilizes mitochondrial function; decreased crystal adhesion	Mittal et al. (2018)
TAE and Arjunic acid	Heart	In vitro	CoCl_2_ induced hypoxia in cardiomyocytes (H9c2)	Downregulation of Bax; upregulated Bcl‐2; Prevents JNK and c‐jun activation; reduced caspase‐3 activation	PMID: 31069430
Self‐assembled arjunolic acid	Breast and cervical cancer	In vitro	MCF‐7 and HeLa cell lines	Reduced GSH and upregulated GSSG levels; ROS generation; elevated pro‐apoptotic TNF‐α and IL‐12; suppressed anti‐apoptotic TGF‐β and IL‐10	Manna et al. (2020)
TA‐AgNPs	Liver, prostate and breast cancer	In vitro	HepG2, PC3, MCF‐7 cancer cells	Upregulated ROS, mitochondrial fragmentation, apoptosis, chromatin condensation and nuclear fragmentation; decreased ΔΨm; disrupted cell integrity; cell cycle arrest	Prasannaraj et al. (2016)
TA‐SeNPs	Breast cancer	In vitro	MCF‐7 cells	Upregulated ROS, ER stress; mitotic arrest, apoptotic bodies, chromosomal instability; Decreased ΔΨm, migration and proliferation	Puri et al. (2023)
Cu/Ag‐MWCNT hybrids	Breast, cervical, liver cancer; Kidney (normal) cells	In vitro	MDA‐MB‐231, HeLa, SiHa, HepG2, Vero cells	Increased ROS, membrane damage, apoptosis, nanoparticle uptake; reduced membrane integrity, proliferation and viability	Yallappa et al. (2016)
Butanolic fraction of alcoholic TAE	Heart	In vivo	Albino Wistar rats with Doxorubicin‐induced acute cardiotoxicity	Free radicle scavenging activity similar to Vit C; Increased SOD, CAT and GSH; decreased lipid peroxidation	Singh et al. (2008)
Hydroalcoholi c *Terminalia arjuna* extract (HETA)	Intestinal epithelial cells (Caco‐2)	In vitro	Amnesic shellfish poisoning	Increased cell viability & membrane potential; restored antioxidant enzyme activity; decreased ROS & NO, apoptosis, nuclear condensation	Ramya et al. (2017)
HETA	Kidney and brain	In vivo	Hypobaric hypoxia‐induced acute mountain sickness (AMS)	Increased GFR and urine output; decreased plasma half‐life of 99mTc‐DTPA and sodium & aldosterone levels; Increased ANP activity antioxidant status, stabilizes electrolyte homeostasis; decreased cerebral vascular leakage; protects renal and cerebrovascular function	Kumar et al. (2016)
HETA	Brain	In vivo	Focal cerebral ischemia–reperfusion injury	Decreased neurological deficits and histopathological damage; Restored SOD, CAT, GSH and decreased MDA & calcium; Preserves Na^+^/K^+^‐ATPase activity	Santhrani (2011)
Alcoholic TAE	Brain	In vivo	Picrotoxin‐induced anxiety	Enhanced antioxidant defense and BDNF/CREB signaling; restored neurotransmitter balance; reduced oxidative stress and improved synaptic plasticity and anxiety‐like behavior, with effects comparable to diazepam	Chandra Sekhar et al. (2017)
α‐tocopherol and HETA	Heart	In vivo	ISO‐induced myocardial damage in Wistar albino rats	Enhanced cellular antioxidants SOD and GSH levels; attenuated high MDA and lipid peroxidation levels; reduced cardiac damage marker levels (SGOT, CK‐MB, Troponin I, and LDH); downregulated inflammatory markers like IL‐6, TNF and CRP; elevated Bax expression; reduced Bcl‐2 levels	Shukla et al. (2015)

#### 4.1.3 Nano‐Engineered *T. arjuna* Formulations in Cancer Therapy

Self‐assembled arjunolic acid (SA‐AA) forms liposome‐like nano‐vesicular structures that selectively target cancer cells while sparing normal cells. In MCF‐7 and HeLa cells, once internalized, SA‐AA disrupts redox homeostasis by depleting glutathione (GSH), elevating glutathione disulfide (GSSG), and increasing ROS and triggering apoptosis. They exert mitochondrial dysfunction while also modulating cytokines by upregulating pro‐apoptotic TNF‐α and IL‐12 and suppressing TGF‐β and IL‐10 (Figure 1). Rescue experiments with ROS scavengers and TNF‐α inhibitors show that its anticancer activity is primarily mediated through a TNF‐α‐driven, ROS‐dependent apoptotic pathway potentially triggering the JNK pathway rather than necrosis (Manna et al. 2020). A direct comparison was made of biosynthesized silver nanoparticles (AgNPs) from *T. arjuna* bark (TaAgNPs) against those from *Plumbago zeylanica* (PzAgNPs) and *Semecarpus anacardium* (SaAgNPs) in HepG2 and PC3 cancer cells. No single source was superior across both cell lines tested—SaAgNPs performed better against HepG2 while TaAgNPs against PC3, causing minimal harm to normal Vero cells at lower doses (Prasannaraj et al. 2016). This suggests that efficacy depends on both the synthesis route and the source plant itself. *Terminalia arjuna* selenium nanoparticles (TA‐SeNPs) showed enhanced cytotoxicity against MCF‐7 cells versus TAE or selenium alone, with ROS‐driven apoptosis and reduced cell migration. TA‐SeNPs reported IC50 (~23.4 μg/mL) substantially higher than doxorubicin's (6.4 μg/mL), meaning TA‐SeNPs were less potent than this standard chemotherapeutic drug, though still considerably more potent than the bark extract (43.62 μg/mL) or selenium alone (69.02 μg/mL) (Puri et al. 2023). TAE‐based copper and silver nanoparticles (Cu‐ and Ag‐MWCNT biohybrids) were prepared using multi‐walled carbon nanotubes, extending the same mechanism across a broader cell line panel (MDA‐MB‐231, HeLa, SiHa, HepG2). It showed reduced viability at higher doses (50 μg/mL; 12%–20%) selective, dose‐dependent cytotoxicity against multiple cancer lines while preserving normal Vero cells (Table 2) (Yallappa et al. 2016).

All four systems point to the same mechanism, and unlike most studies here, the AgNP study directly compared *T. arjuna* against other plant sources rather than testing it alone. But none have been tested for pharmacokinetics, toxicity, or safety in living organisms, and none have a scalable production method, which is an important future direction.

### 4.2 Cardiovascular Protection by *T. arjuna*

#### 4.2.1 Anti‐Apoptotic Protection Against Cardiotoxicity

Across three different toxic‐injury models, including cobalt chloride‐induced hypoxia, doxorubicin‐induced cardiotoxicity, and sodium nitrite‐induced injury, TAE and its constituents consistently protect cardiomyocytes through a shared antioxidant, anti‐apoptotic mechanism, though each model reflects a distinct clinical scenario. Hypoxiamimetic agents such as cobalt chloride (CoCl_2_) generate ROS ‐and trigger oxidative stress, apoptosis, and cardiac injury (Wang et al. 2016). In H9c2 cardiomyocyte cells, CoCl_2_ reduces viability and increases LDH release, depletes antioxidant enzymes (SOD, CAT, GPx, GR, GST), glutathione (GSH) with upregulated stress‐responsive HSP70. TAE, arjunic acid pretreatment restores viability, attenuates LDH leakage, with normal HSP70 levels, along with modulating key apoptotic proteins by downregulating Bax, suppressing JNK and c‐jun phosphorylation. There this is followed by caspase‐3 inhibition and upregulating anti‐apoptotic Bcl‐2 members, as shown in Figure 2 (Manu et al. 2019). Doxorubicin is a potent antineoplastic agent used in the treatment of various solid tumors, but its clinical utility is limited by cumulative cardiotoxicity and the generation of ROS and reactive nitrogen species (RNS) (Carvalho et al. 2009). In male Wistar rats, oral administration of the butanolic fraction of alcoholic TAE conferred cardioprotective effects. Pre‐treatment with this extract demonstrated strong free radical scavenging activity in vitro, comparable to vitamin C and elevated endogenous antioxidants (SOD, CAT, GSH), decreased lipid peroxidation, reduced ROS‐mediated organ damage (Singh et al. 2008). Another study highlights AA as a cytoprotective agent against doxorubicin‐induced cardiotoxicity, promoting apoptotic cardiomyocyte death. AA attenuated increased p53 activation; treatment with the p53 inhibitor Pifithrin‐α (PFT‐α) abrogates doxorubicin‐mediated cell death, confirming p53's central role (B.‐J. Kim et al. 2006). In doxorubicin‐exposed cardiomyocytes, AA suppresses p38/JNK‐driven Bax translocation, preserves mitochondrial membrane potential, prevents cytochrome c release, and stops the Bcl‐2/Bcl‐xL downregulation that doxorubicin normally causes (Ghosh et al. 2011). Because doxorubicin remains an actively used chemotherapy agent whose cardiotoxicity limits its clinical use, this protective effect raises a possibility that *T. arjuna* can be a cardioprotective adjunct for patients undergoing chemotherapy, connecting this finding back to the anticancer evidence discussed in Section 3. Sodium nitrite exposure causes diseases like leukemia, cancer, tumors, nephrotoxicity and hepatotoxicity (Salama et al. 2013). AA mitigates sodium nitrite‐induced cardiac injury by reversing oxidative stress markers (elevated MDA, depleted GSH), restoring mitochondrial cytochrome c oxidase activity, and normalizing pro‐inflammatory cytokines (TNF‐α, IL‐1β) while increasing anti‐inflammatory IL‐4 and IL‐10. Sodium nitrite‐treated hearts show activated JNK and cleaved caspases‐3, −8, and −9, indicating both extrinsic and intrinsic apoptotic pathways; AA treatment suppresses JNK activation, cytokine imbalance, and caspase cleavage, thereby limiting both oxidative‐driven and inflammatory‐mediated apoptosis (Al‐Gayyar et al. 2014).

**FIGURE 2:**
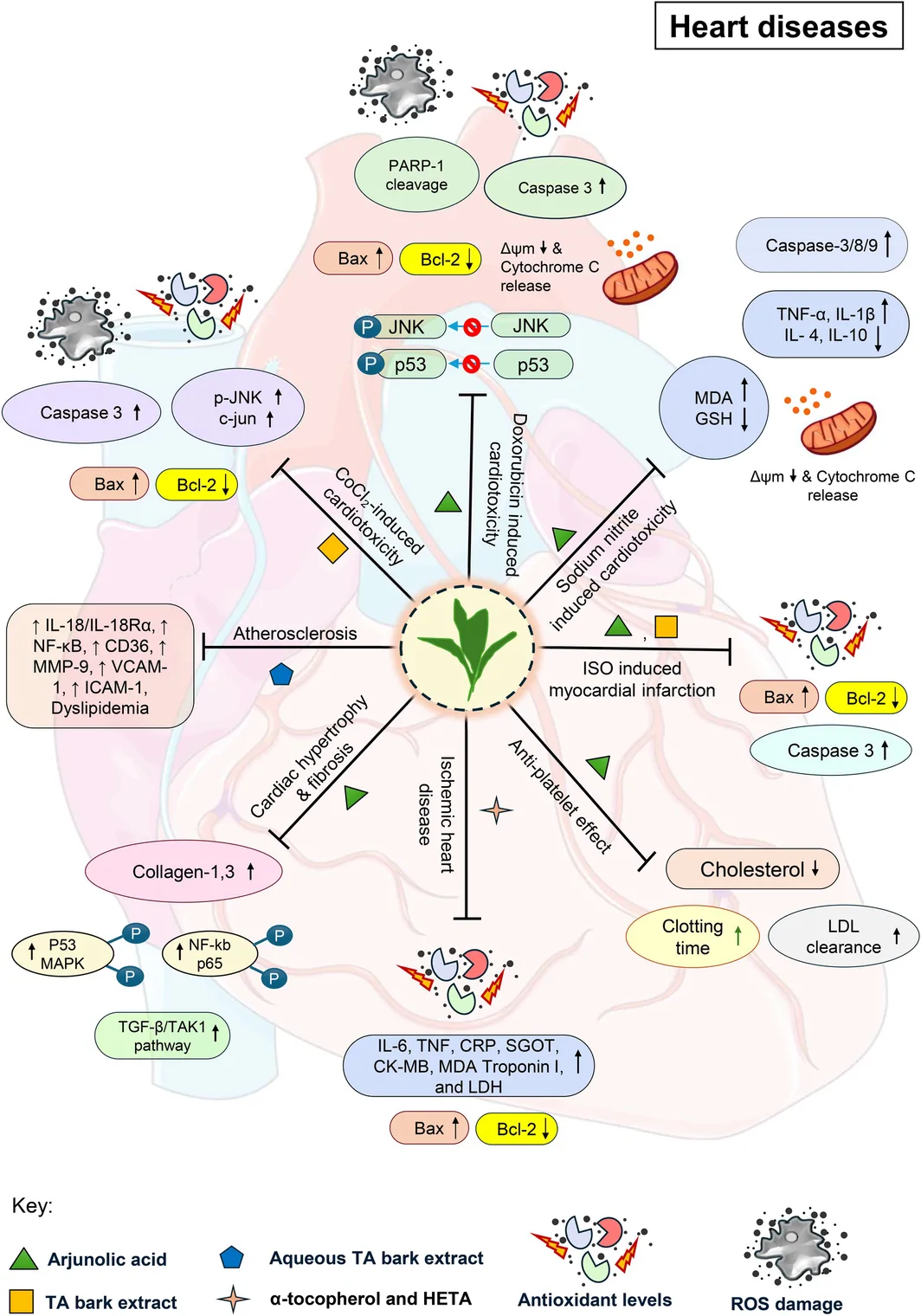
Cardioprotective mechanisms of *Terminalia arjuna* and its bioactives against heart diseases. *Terminalia arjuna* and its active compounds (arjunolic acid, arjunic acid, arjungenin, and arjunetin) protect against various cardiac pathologies including atherosclerosis, fibrosis, hypertrophy, and drug‐ or toxin‐induced cardiotoxicity. The treatments reduce oxidative stress (ROS, MDA), apoptosis (Bax, caspase‐3/8/9, PARP‐1 cleavage), and inflammation (TNF‐α, IL‐1β, NF‐κB), while enhancing antioxidant defenses (GSH) and anti‐apoptotic signaling (Bcl‐2). Additionally, *T. arjuna* modulates key pathways including JNK/p53, TGF‐β/TAK1, and NF‐κB, contributing to improved cardiac remodeling and myocardial protection.

#### 4.2.2 Antioxidant and Anti‐Ischemic Actions

ISO, a known synthetic catecholamine and β‐adrenergic agonist, is commonly used to induce myocardial infarction and myocardial necrosis in animal models (Yousefi et al. 2013). Consistent with the toxin‐injury studies above, ISO‐induced myocardial necrosis is characterized by depletion of antioxidant enzymes (SOD, CAT, GPx, GR) and GSH, elevated free radicals, and lipid peroxidation leading to irreversible membrane damage. Pre‐ and post‐treatment with AA elevates antioxidant enzyme activities, reduces lipid peroxidation, and normalizes myeloperoxidase activity, thereby limiting oxidative injury and neutrophil‐mediated inflammation (Sumitra et al. 2001). Similarly, TAE protects against ISO‐induced oxidative cardiac stress just like AA. TAE also suppresses iNOS‐mediated NO overproduction, blocks JNK/c‐jun signaling, and reduces caspase‐3 activation, while targeting Bcl‐2/Bax, highlighting its anti‐hypertrophic effects (Thangaraju et al. 2020).

Ischemic heart disease (IHD) ranks among the top causes of mortality in industrialized countries, often manifesting first as myocardial infarction in the majority of patients (Manfroi et al. 2002). *T. arjuna* has served as a traditional remedy for heart conditions since ancient times, while α‐tocopherol (Vitamin E) acts as a potent antioxidant and free radical scavenger (Smolarek and Suh 2011). Hydroalcoholic extract of *T. arjuna* (HETA) combined with α‐tocopherol (vitamin E) markedly enhances endogenous antioxidant defenses (SOD, GSH) and blunts ISO‐induced increases in MDA, cardiac enzymes (SGOT, LDH, CK‐MB, troponin I), along with inflammatory markers (IL‐6, TNF‐α, CRP). HETA plus α‐tocopherol also confirms Bax/Bcl‐2 dysregulation, mitigating apoptosis‐driven myocyte loss (Shukla et al. 2015).

#### 4.2.3 Anti‐Atherogenic and Lipid‐Modulating Effects

Atherosclerosis is a chronic inflammatory disease which arises from endothelial injury, LDL‐c oxidation, and macrophage foam‐cell formation via scavenger receptors (SR‐A, SR‐B1, CD36) (Alfarisi et al. 2020; Poznyak et al. 2021). Nuclear receptors including PPARs—α, δ, γ and LXR α regulate transcription of genes involved in lipid metabolism, cholesterol efflux, and inflammation in macrophages, smooth muscle cells, etc., influencing disease progression (Li and Glass 2004). IL‐18, a key pro‐inflammatory cytokine, is activated via the inflammasome, promotes atherosclerosis by driving NF‐κB and p38 MAPK mediated inflammation, promoting endothelial dysfunction, and plaque progression, with elevated levels linked to plaque instability and rupture (Hoshino et al. 1999). IL‐18–treated apolipoprotein E‐knockout (ApoE−/−) mice induced atherosclerosis showing upregulated IL‐18/IL‐18Rα and NF‐κB expression, increased CD36‐mediated lipid uptake and foam cell formation, elevated matrix metalloproteinase‐9 (MMP‐9), vascular cell adhesion molecule‐1 (VCAM‐1), and intercellular adhesion molecule‐1 (ICAM‐1) levels, dyslipidemia and extensive plaque development. Treatment with aqueous TAE downregulates IL‐18/IL‐18Rα and NF‐κB, suppresses pro‐atherogenic markers (CD36, MMP‐9, VCAM‐1, ICAM‐1), and activates cholesterol‐efflux regulators PPAR‐γ and LXR‐α, leading to reduced total cholesterol, LDL‐c, triglycerides, and plaque burden with improved HDL‐c and aortic‐lesion distribution. The study reports these effects as comparable to atorvastatin; however, this comparison comes from a single animal study rather than a head‐to‐head trial designed to test relative safety (Bhat et al. 2018).

#### 4.2.4 Cardiac Hypertrophy & Fibrosis

Cardiac hypertrophy and fibrosis, often driven by pressure overload and TGF‐β‐mediated extracellular matrix remodeling, impair myocardial function and predispose to heart failure (Kong et al. 2014). AA exhibits antioxidant, anti‐inflammatory, anti‐fibrotic properties across various cardiac disease models (Ghosh and Sil 2013; Maulik and Talwar 2012). In the present study, AA was identified as an agonist of PPARδ, binding directly to its ligand‐binding domain and stabilizing its active conformation. Through this interaction, AA enhanced PPARδ expression in an autoregulatory loop and mediated direct inhibition of TAK1 via its N‐terminal AF‐1 domain, thereby blocking non‐canonical TGF‐β signaling pathways (NF‐κB, p65, p38 MAPK) responsible for extracellular matrix protein overproduction (Guo et al. 2004; Subramanian et al. 2017), while sparing canonical SMAD signaling. This dual role as a PPARδ activator and TAK1 suppressor underlies AA's ability to regress cardiac fibrosis and improve myocardial function during hypertrophy. In Angiotensin II‐stimulated cardiac fibroblasts and renal artery ligation models, AA reduced collagen‐1 and collagen‐3 expression, lowered myocardial collagen content, and improved echocardiographic parameters. Functional knockdown experiments confirmed that PPARδ is critical to AA's anti‐fibrotic effects; silencing PPARδ in hypertrophied cells and animal models eliminated AA's capacity to decrease collagen synthesis, confirming PPARδ as the mechanism's central node rather than an incidental correlate (Bansal et al. 2017).

#### 4.2.5 Anti‐Platelet Effect

Platelet agonists like thrombin, ADP, collagen, and TXA_2_ can trigger activation and GP IIb/IIIa‐mediated aggregation (Yun et al. 2016). Aspirin, a standard antiplatelet therapy, inhibits this process, reducing the risk of coronary reocclusion and reinfarction (Wang et al. 2025). AA exhibits potent antiplatelet activity (IC_50_ 0.048 ± 0.014), outperforming aspirin (0.088 ± 0.017 mM), highlighting its potential as an antiplatelet agent. This in vitro result is a promising pharmacological signal, but it does not establish clinical efficacy comparable to aspirin, whose safety profile is supported by decades of clinical use that AA has not yet undergone. Beyond platelet inhibition, AA demonstrates hypocholesterolemic effects by reducing hepatic cholesterol synthesis, increasing bile acid excretion, and enhancing LDL clearance. It prolongs clotting time, indicating anticoagulant activity and broad cardiovascular protection (Sumitra et al. 2001).

Across all the studies, *T. arjuna* consistently protects the heart through antioxidant and anti‐apoptotic action against toxins, ischemia, atherosclerosis, fibrosis, and platelet activation alike. Together with evidence from clinical trials (discussed in a later section), cardiovascular protection stands out as the most concrete evidence among *T. arjuna's* therapeutic effects.

### 4.3 Liver‐Protective Effects of *T. arjuna*

#### 4.3.1 Anti‐Apoptotic and Anti‐Necrotic Hepatoprotection

Acetaminophen (N‐acetyl‐p‐aminophenol, APAP) is widely used for pain and fever but can cause dose‐dependent hepatotoxicity and hepatic necrosis due to overuse (Ayoub 2021). AA protects against APAP‐induced liver injury mainly by inhibiting cytochrome P450 enzymes (CYP's), particularly CYP2E1, which otherwise convert APAP into the toxic metabolite N‐acetyl‐p‐benzoquinone imine (NAPQI). NAPQI binds to mitochondrial proteins and GSH, initiating lipid peroxidation, oxidative stress, and mitochondrial dysfunction (Ramachandran and Jaeschke 2019). AA pretreatment preserves GSH, scavenges superoxide, hydroxyl, and NO radicals, and suppresses hydrogen peroxide, thereby mitigating oxidative stress‐mediated hepatotoxicity along with JNK activation, cytochrome c release, promotes mitochondrial dysfunction (ΔΨm loss), blocking both apoptotic and necrotic cell death (Ghosh et al. 2010b). Similarly, in cadmium chloride (CdCl_2_) exposed hepatocytes, CdCl_2_ induces apoptosis through both intrinsic (Bax/Bak, cytochrome c, caspase‐9/3) and extrinsic (Fas, caspase‐8) pathways, along with NF‐κB‐mediated inflammation. AA preserves mitochondrial integrity, suppresses these apoptotic markers, and inhibits NF‐κB activation, thereby protecting hepatocytes from oxidative and inflammatory injury (Pal et al. 2011).

Across APAP and cadmium induced injury models, AA protects hepatocytes by preserving mitochondrial integrity and suppressing both intrinsic and extrinsic apoptotic pathways. However, these findings are based on experimentally induced toxicity, whether this protection is effective in chronic liver diseases in humans such as viral hepatitis or fatty liver disease is unclear.

#### 4.3.2 Antioxidant Mediated Hepatoprotection

The antioxidant effects of AA have also been demonstrated in arsenic, cadmium, and fluoride induced hepatotoxicity. In sodium arsenite (NaAsO_2_) exposed hepatocytes, alanine transaminase (ALT) and alkaline phosphatase (ALP) rise, indicating membrane leakage and oxidative injury. FRAP and antioxidant enzymes (SOD, CAT, GST, GR, GPx) decline, GSH and total thiols drop, while ROS and GSSG increase, reflecting redox imbalance. AA restores antioxidant power, upregulates SOD, CAT, GST, GR, GPx, and total thiols, decreases GSSG, and normalizes ALT/ALP, acting as a potent free‐radical scavenger similar to vitamin C. Its dual action in direct radical scavenging (via hydroxyl and carboxyl groups) and arsenic chelation underlies AA's protection against arsenic‐induced oxidative hepatotoxicity (Manna, Sinha, Pal, and Sil 2007). A parallel study in Swiss albino mice showed that AA counters arsenic‐induced testicular oxidative stress, restoring SOD, CAT, GST, GR, GPx, GSH, and total thiols while reducing GSSG, ROS/RNS, lipid peroxidation, protein carbonylation, and histological damage, reinforcing its broad‐spectrum antioxidant and cytoprotective profile (Manna et al. 2008). In Swiss albino mice, AA reduced CdCl_2_‐induced oxidative liver injury by restoring GSH/GSSG balance and antioxidant enzymes while lowering MDA and protein carbonylation (Pal et al. 2011). Similarly, in murine hepatocytes, AA protected against NaF‐induced oxidative stress and necrosis by restoring antioxidant defenses and reducing lipid and protein damage, with effects comparable to vitamin C (Ghosh et al. 2008).

Taken together, the arsenic, cadmium, and fluoride hepatotoxicity studies show a consistent antioxidant effect of AA, but the evidence is limited by the variable doses (20 to 100 mg/kg), lack of dose–response studies, and comparison mainly with vitamin C rather than established hepatoprotective drugs. None of the studies have been extended to exposure in humans, so this antioxidant signal is not yet matched in the clinics.

### 4.4 Reno‐Protective Effects

In isolated perfused rat kidneys, TAE reduced oxidative injury by enhancing glutathione‐dependent antioxidant defenses and limiting membrane damage (Raj et al. 2012). AA counters cisplatin‐induced renal injury by reversing oxidative stress via NADPH oxidase‐mediated ROS, restores antioxidant defenses and reduces lipid peroxidation (El‐Sawalhi and Ahmed 2014; Kim et al. 2010). Cisplatin also activates NF‐κB and upregulates pro‐inflammatory cytokines (TNF‐α, IL‐1β), AA suppresses these mediators, lowers TGF‐β and TGF‐βR1, and inhibits caspase‐3, −8, and −9 activation, thus limiting both inflammatory and apoptotic pathways (Elsherbiny et al. 2016). These findings converge into a protective mechanism where AA limits the oxidative stress, inflammation, apoptosis rather than acting through a single pathway. In parallel work, AA administration reduces cisplatin‐induced nephrotoxicity by enhancing renal GSH, lowering MDA and NO, normalizing NF‐κB activity, TGF‐β expression, and kidney injury molecule‐1 (Kim‐1) (Sherif 2015). As noted earlier (Ghosh et al. 2010b), AA protects against APAP‐induced hepatotoxicity which significantly reduced necrotic cell death in liver. In a related study a similar pattern was observed in APAP induced renal injury, where AA restored antioxidant defenses and mediated caspase dependent responses. APAP‐triggered renal inflammation which elevated TNF‐α, iNOS‐derived NO, and peroxynitrite formation, all of which are curbed by AA via TNF‐α‐driven iNOS downregulation (Ghosh et al. 2010a).

Many evidences also extend to renal stone diseases, which causes recurrent morbidity and tissue damage via calcium oxalate monohydrate (COM) crystal formation (Alelign and Petros 2018). Aqueous TAE (aqTAE) reduces calcium oxalate crystallization and crystal adhesion to tubular epithelial cells while limiting oxidative and apoptotic injury (Mittal et al. 2017). Similar results were concluded in the study by (Mittal et al. 2016a). Oxalate‐induced apoptosis characterized by nuclear condensation and fragmentation, and elevated caspase‐3 was reversed by TAE treatment, likely by stabilizing mitochondrial function and suppressing ROS‐driven apoptosis (Figure 3) (Mittal et al. 2018). From *T. arjuna* bark, four novel anionic proteins‐nuclear pore anchor (A1), DEAD‐box RNA helicase 45 (B1), Lon protease‐1 (B2), and HSP 90–3 (C1) have been identified which have similarly demonstrated antiurolithiatic and cytoprotective effects (Mittal et al. 2016b). Together they complement the phytochemical profile of *T. arjuna* to demonstrate multifaceted renoprotection, while further studies can help to identify the key components responsible for this phenotype.

**FIGURE 3:**
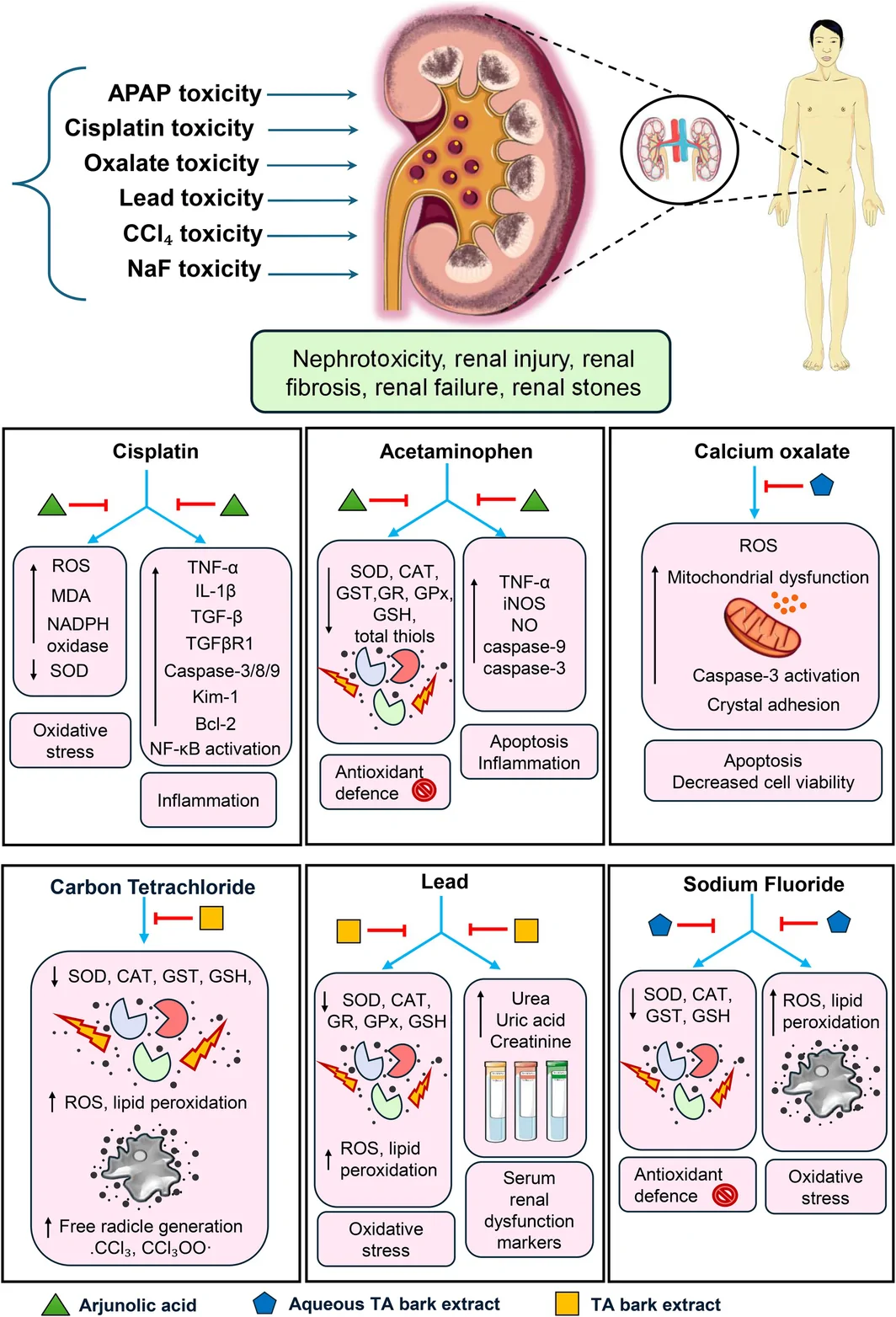
Nephroprotective mechanisms of *Terminalia arjuna* against toxin‐induced renal injury. *Terminalia arjuna* bark extract and its bioactive compounds like AA protect against nephrotoxicity caused by cisplatin, APAP, CaOx, CCl_4_, LA, and NaF. The treatments mitigate oxidative stress, apoptosis, and inflammation by enhancing antioxidant enzymes (SOD, CAT, GST, GR, GPx, GSH) and reducing lipid peroxidation, TNF‐α, IL‐1β, and caspase activation. TA also restores renal function markers (urea, creatinine, uric acid) and prevents mitochondrial dysfunction, preserving renal structure and function.

#### 4.4.1 Multi‐Organ Antioxidant Actions of *T. arjuna*

Across multi‐organ oxidative stress models, aqTAE protects liver and kidney against carbon tetrachloride (CCl_4_), a classic hepatotoxin that generates free radicals via CYP‐mediated activation, initiating lipid peroxidation and membrane damage (Boll et al. 2001; Ganie et al. 2011). In CCl_4_‐treated mice, aqTAE pretreatment restores antioxidant enzymes and GSH, reduces lipid peroxidation, and scavenges DPPH radicals similarly to vitamin C, limiting tissue damage (Manna et al. 2006; Manna, Sinha, and Sil 2007). The findings are further supported by sodium fluoride (NaF)‐induced toxicity in mice liver and kidney where aqTAE (40 mg/kg for 10 days) pretreatment normalizes SOD, CAT, GST, and GSH levels and shows potent DPPH‐scavenging, implicating oxidative stress as a central mechanism of fluoride‐driven organ toxicity (Sinha et al. 2007). Similarly, in Swiss albino mice, TAE (75 mg/kg) also reduced lead acetate induced oxidative damage by restoring antioxidant defenses and GSH levels, establishing *T. arjuna* as a broad‐spectrum antioxidant agent for mitigating heavy‐metal‐induced organ injury (Table 2) (Mohammed Raouf et al. 2013). These studies suggest that the protective effect of TAE is not restricted to a single toxicant or organ but has a broader capacity to preserve homeostasis under stressful conditions. Future studies incorporating proper dose response experiments can determine whether these antioxidant effects are reproducible beyond animal models.

### 4.5 Wound Healing

Wound healing is an essential biological process which protects the skin by preserving its integrity post trauma (Rodrigues et al. 2019) and is enhanced by natural products with medicinal properties such as anti‐inflammatory, antioxidant, antibacterial, pro‐collagen synthesis, and fight infection which speed up wound healing (Ibrahim et al. 2018; Ti et al. 2022). Phytochemicals such as tannins, alkaloids, terpenoids, and saponins modulate key phases of healing, including inflammation, granulation, and remodeling (Thakur et al. 2011). Bioactive compounds of *T. arjuna* have been evaluated for the wound healing activity as it reduces inflammation in vessels, heals ulcers, and increases tensile strength of wounds, positioning it as a promising phyto‐nutraceutical and cosmeceutical agent (Pratap Shankar and Ashwathykutty 2024). In rat models, hydroethanolic bark extract (HETA) enhanced granulation tissue formation and hydroxyproline content, indicating increased collagen deposition and tissue repair (Rane and Mengi 2003). Phytochemical analysis of the extract identified tannins, which were associated with the most pronounced wound healing activity in fraction I. This fraction also significantly increased the tensile strength of incision wounds and accelerated epithelialization in excision wound models compared to fractions II and III (with saponins and reducing sugars, and mainly saponins respectively), which even outperformed the standard reference agent, Betadine. The enhanced epithelialization is attributed to the astringent nature of tannins, which facilitate tissue contraction and closure. Biochemical assessment of granulation tissue revealed a significant increase in hexosamine content with fraction I, indicating it helps stabilize collagen molecules to enhance epithelialization and provides strength to repair the tissue (Nayak et al. 2009). In contrast, Fractions II and III did not produce statistically significant changes in hexosamine levels, likely due to the presence of reducing sugars, which are known to delay wound repair processes. The same fraction also showed antibacterial activity against *Pseudomonas aeruginosa* , *Escherichia coli* , *Staphylococcus aureus* , and *Streptococcus pyogenes* , but not *Candida albicans* which contribute to its wound healing efficacy by reducing the risk of secondary infections, a common complication in wound sites (Tables 1 and 3) (Chaudhari and Mengi 2006). Overall, these data underscore *T. arjuna*, particularly its tannin‐rich fractions, as a multifunctional phyto‐nutraceutical candidate for topical or systemic wound‐care formulations, with potential for integration into functional foods, nutraceuticals, and cosmeceutical products targeting tissue repair and skin barrier integrity.

**TABLE 3:** Summary of the antimicrobial, wound‐healing, and antidiabetic effects of *Terminalia arjuna* extracts.

Extract/metabolite	Key phytochemicals	Experimental system/targets/model	Experimental findings	Outcome/effect	References
Methanolic bark extract	—	Gram‐positive: *Staphylococcus aureus* ; *Streptococcus mutans* ; Gram‐negative: *Escherichia coli; Klebsiella pneumoniae*	Strong antimicrobial activity; zone of inhibition strongest against Gram‐negative bacteria	Strong antimicrobial activity	Mandal et al. (2013)
Methanolic extract of *Terminalia arjuna* (with *Camellia sinensis* )	—	Gram‐positive: *Staphylococcus aureus* ; Gram‐negative: *Pseudomonas aeruginosa* ; *Escherichia coli*	Max activity against Candida > *E. coli* > *P. aeruginosa* > *S. aureus*	Antimicrobial activity	Gupta and Kumar (2017)
*Terminalia arjuna* extract	—	Gram‐positive: *Bacillus subtilis* ; Gram‐negative: *Escherichia coli; Pseudomonas aeruginosa; Raoultella planticola*	Bound and free flavonoids showed antimicrobial activity against different pathogenic bacteria	Antimicrobial activity	Jaiswal and Kumar (2016)
n‐Butanol fraction (phenolic‐rich)	Phenolics and flavonoids	Gram‐positive: *Bacillus subtilis; Staphylococcus aureus*; Gram‐negative: *Escherichia coli; Klebsiella pneumoniae; Pseudomonas aeruginosa; Salmonella typhi*	Highest antibacterial & strong antioxidant activity; rich in phenolics and flavonoids hence indicated increased antioxidant activity	Antibacterial and antioxidant activity	Kumar et al. (2018), Kumar et al. (2021)
Acetone extract	—	Gram‐positive: *Staphylococcus aureus* ; Gram‐negative: *Acinetobacter; Pseudomonas aeruginosa; Proteus mirabilis*; Fungal: *Candida albicans*	Strong antibacterial (zones 16–28 mm), no antifungal effect	Strong antibacterial activity	Dhiman et al. (2016)
Casuarinin	—	African green monkey kidney cells (Vero cells); herpes virus	Shows strong anti‐herpes activity; no cytotoxicity toward Vero cells at effective concentrations	Anti‐herpes activity	Cheng et al. (2002)
Hydroalcoholic bark extract	Tannins, saponins, reducing sugars	Rat dermal wound models	Increased granulation tissue, elevated hydroxyproline	Faster collagen turnover; rapid wound healing	Rane and Mengi (2003)
Fraction I (tannin‐rich)	Tannins	Incision wounds; *P. aeruginosa, E. coli, S. aureus, S. pyogenes*	Highest tensile strength in incision wounds (better than Betadine); complete epithelial closure by Day 9 (vs. Day 18 with Betadine); increased hexosamine in granulation tissue; antimicrobial activity against *P. aeruginosa* , *E. coli* , *S. aureus*, *S. pyogenes*	Strong wound contraction & closure; faster epithelialization; stabilized collagen molecules; prevents infection at wound site	Chaudhari and Mengi (2006)
Fraction II (saponins + reducing sugars)	Saponins + reducing sugars	Wound model	No significant increase in hexosamine	Limited wound healing effect	Chaudhari and Mengi (2006)
Fraction III (mainly saponins)	Saponins	Wound model	No significant improvement in tensile strength or hexosamine	Weak wound healing activity	Chaudhari and Mengi (2006)
General bark effects	—	—	Reduces inflammation in vessels, heals ulcers, improves tensile strength	Supports wound healing activity overall	Pratap Shankar and Ashwathykutty (2024)
TAE	—	Glucose/metabolic system; specific model not stated in supplied table	Reduces blood glucose and HbA1c; improves glucose tolerance, hepatic glycogen; increases insulin hormone & C‐peptide	Strong glycemic improvement; increases insulin availability	Aamir et al. (2022)
Arjunic acid/TAE	—	Insulin‐signaling/inflammatory pathways	Increases IRS‐1, PI3K/Akt signaling; reduces JNK1/2 and restored IRS‐1; inhibits TLR‐4/MyD88/NF‐κB; downregulates TNF‐α, IL‐6, IL‐1β	Improved insulin signaling, glucose uptake; reduces cytokine injury and apoptosis; anti‐inflammatory effect	Aamir et al. (2023)
TAE	—	Glucose/metabolic system; specific model not stated in supplied table	Reduces blood glucose and increases glucose tolerance	Improved glycemic control	Biswas, Kar, et al. (2011)
Arjunic acid	—	Oxidative stress/mitochondrial and apoptosis pathways	Reduces ROS and lipid peroxidation; stabilizes mitochondria and regulates Bcl‐2 proteins; prevents PARP‐mediated NAD+/ATP loss	Antioxidant protection; anti‐apoptotic effect	Manna, Das, et al. (2010)
Arjunic acid	—	Antioxidant and inflammatory signaling; kidney	Restores SOD, CAT, GPx, GSH/GSSG; inhibits NF‐κB, ERK, p38, JNK	Reverses redox imbalance; anti‐inflammatory effect; prevents kidney damage	Manna et al. (2009a), Manna et al. (2009b)
TAE/arjunic acid	—	Immune signaling	Upregulates IL‐2 and IFN‐γ; downregulates TNF‐α	Restores immune balance	Manna, Ghosh, et al. (2010)
Arjunic acid/TAE	—	Glucose, lipid and inflammatory/cardiac markers	Reduces blood glucose; improves hepatic glycogen; lowers CK, LDH, CK‐MB; lowers NF‐κB, MAPKs; reduces cholesterol, LDL, TG and increases HDL	Glycemic improvement; cardioprotection; anti‐inflammatory effect; normalizes lipid profile	Manna and Sil (2012a), Manna and Sil (2012b)
Arjunic acid, arjungenin, Rutin, quercetin, ellagic acid, gallic acid	—	DPP‐IV & digestive enzymes	High binding affinity with DPP‐IV & digestive enzymes	Stronger than standard drugs	Tahsin et al. (2021)
TAE	—	DPP‐IV	Potent DPP‐IV inhibition	Comparable to sitagliptin	Mohanty et al. (2019)
TAE	—	α‐amylase & α‐glucosidase	Inhibits α‐amylase & α‐glucosidase	Reduces postprandial glucose	Saha and Verma (2012)
TAE/arjunic acid	—	PGC‐1α; glucose uptake & mitochondria; lipid metabolism	Increases PGC‐1α (glucose uptake & mitochondria); reduces TGs, increases HDL	Improved energy metabolism; improved lipid profile	Shengule et al. (2018)
Arjunic acid	—	AMPK‐mTOR‐HO‐1 autophagy pathway	AMPK‐mTOR‐HO‐1 autophagy activation	Decreases oxidative stress	Zhang et al. (2022)
TAE	—	Cardiac inflammation; VEGF, ICAM‐1, MCP‐1; LV function and autonomic regulation	Reduces TNF‐α, IL‐6, IL‐1β; increases VEGF, ICAM‐1, MCP‐1; preserves LV function & autonomic regulation	Decreases cardiac inflammation	Khaliq, Parveen, Singh, Gondal, et al. (2013); Khaliq, Parveen, Singh, Hussain, and Fahim (2013)

### 4.6 Infectious Diseases

#### 4.6.1 Anti‐Bacterial and Anti‐Fungal

*T. arjuna* demonstrates broad inhibitory effects on various microorganisms in vitro. The methanolic extract of the bark revealed many flavonoids synthesized in response to microbial infection releasing antimicrobial substance likely due to the ability of these compounds to complex with extracellular and soluble proteins and with bacterial cell wall (Cowan 1999). The methanolic bark extract of *T. arjuna* demonstrated antimicrobial activity against human pathogenic bacteria, including Gram‐positive ( *Staphylococcus aureus* , *Streptococcus mutans* ) and Gram‐negative ( *Escherichia coli* , *Klebsiella pneumoniae* ), with inhibition comparable to the standard drug chloramphenicol. The highest zones of inhibition were observed against two Gram‐negative bacteria (Mandal et al. 2013). In a study, *Camellia sinensis* and *T. arjuna* plant extract were used to study the antimicrobial effects against *Staphylococcus aureus, Pseudomonas aeruginosa, Escherichia coli* and *Candida albicans* . Test results showed antibacterial activity against all the microorganisms; *T. arjuna* exhibited maximum antimicrobial activity against *Candida albicans* , followed by *Pseudomonas aeruginosa, Escherichia coli* and *Staphylococcus aureus* (Gupta and Kumar 2017). These results were in line with (Jaiswal and Kumar 2016) where both bound and free flavonoids showed antimicrobial activity against different pathogenic bacteria *Escherichia coli* , *Pseudomonas aeruginosa* , *Raoultella planticola* , *Bacillus subtilis* . This activity varied with the extraction method and phytochemical composition, as phenolic and flavonoid rich fractions, particularly the n‐butanol fraction, generally show stronger antibacterial effects than other extracts. The n‐butanol fraction exhibited more antibacterial activity compared to the ethyl acetate and ethanolic extract which was tested against *Bacillus subtilis, Staphylococcus aureus, Escherichia coli, Klebsiella pneumoniae, Pseudomonas aeruginosa, Salmonella typhi* (V. Kumar et al. 2018). This was confirmed by a similar study performed by (V. Kumar et al. 2021). The acetone extract of *T. arjuna* showed strong antibacterial effect against *S. aureus* , *Acinetobacter*, *P. aeruginosa* , and *Proteus mirabilis* , compared to the methanolic, ethanolic, and aqueous extracts. However, it showed no activity against *Candida albicans* (Dhiman et al. 2016). These findings highlight the strong antimicrobial and antioxidant potential of the methanolic extract of *T. arjuna* (Table 3).

#### 4.6.2 Herpes Simplex Virus Anti‐Viral Activity

Herpes simplex virus Type 1 (HSV‐1) and HSV‐2 are common herpesviruses causing oral and genital infections, respectively, that particularly affect immunocompromised patients (Hou et al. 2017). Although acyclovir is widely used for HSV management, frequent use has led to the development of acyclovir‐resistant strains in the clinic (Schalkwijk et al. 2022). Casuarinin, isolated from the bark of *T. arjuna*, shows anti‐HSV‐2 activity in vitro, with IC_50_ values of 3.69 ± 0.9 mM and 1.59 ± 0.2 mM with no cytotoxicity toward Vero cells. Mechanistically, casuarinin interferes with HSV‐2 attachment and penetration by disrupting viral glycoproteins, particularly glycoprotein C and glycoprotein D. This suggests casuarinin inhibits infection by blocking the initial stages of viral entry. Experimental assays confirmed Casuarinin does not directly destroy the virus and remains active even when added 12 h after infection, suggesting it may also affect later stages of HSV‐2 replication. These results highlight casuarinin's anti‐viral potential, but the lack of in vivo data limits its credibility (Cheng et al. 2002).

### 4.7 DNA Damage

Domoic acid toxicosis causes amnesic shellfish poisoning and neuroexcitatory effects like seizures and memory loss (McKibben et al. 2017; Ramya et al. 2022). In Caco‐2 cells, domoic acid reduced viability, triggered mitochondrial dysfunction, ROS/NO overproduction, antioxidant enzyme depletion, and apoptosis. HETA pretreatment restored viability, morphology, membrane potential, antioxidant enzyme activity, and redox balance while curbing nuclear damage (Table 2). LC‐ESI–MS/MS analysis of HETA revealed the presence of several phytoconstituents like AA, arjungenin, kaempferol 3‐glucuronide, ursolic acid, ellagic acid, etc. Among other phytocompounds tested, kaempferol and arjungenin also demonstrated protective effects, improving cell viability and adherence properties, reducing ROS and NO generation, and mitigating apoptotic changes, comparable to HETA. In contrast, ellagic acid, ursolic acid, and AA did not show marked protective effects. Together, these findings suggest that HETA exerts potent cytoprotective and antioxidant effects by maintaining redox homeostasis and mitigating apoptotic cell death (Ramya et al. 2017).

### 4.8 Neuroprotective Activity

Preclinical studies suggest that *T. arjuna* protects against different forms of neurological injury, largely through antioxidant, anti‐inflammatory, and vascular mechanisms. In rats with cerebral ischemia, aqTAE (500 mg/kg/day) reduced infarct size, neurological deficits, limits vascular leakage and BBB leakage by restoring tight‐junction proteins, suppressing MMP‐2/9 and COX‐2, and upregulates TIMP‐1/2 (Table 1) and reducing oxidative stress (Kaliyappan et al. 2021). Similarly, in transient middle‐cerebral‐artery‐occlusion (MCAO)–induced cerebral ischemia/reperfusion rat models, alcoholic TAE and AA‐enriched fractions reduced neurological damage and oxidative stress while preserving GSH, SOD, catalase, Na^+^/K^+^‐ATPase, and mitochondrial function (Santhrani 2011; Yaidikar and Thakur 2015). These findings suggest that *arjuna* may protect the ischemic brain by maintaining redox and mitochondrial homeostasis. At high altitude, hypoxia can trigger acute mountain sickness (AMS) in non‐acclimatized individuals by disrupting renin‐angiotensin‐aldosterone system (RAAS) signaling, causing fluid imbalance, renal dysfunction, and cerebrovascular leakage (Loeppky et al. 2003). Excessive RAAS activation drives sodium retention and blood–brain barrier disruption, while atrial natriuretic peptide (ANP) counteracts this by promoting vasodilation, diuresis, and RAAS suppression (Kuwahara 2021). In rats exposed to acute hypobaric hypoxia (27,000 ft), vehicle‐treated animals showed reduced GFR, prolonged technetium‐99 m DTPA half‐life, low urine output, RAAS hyperactivation, suppressed ANP, and increased cerebral vascular leakage. HETA pretreatment reversed these effects, restoring GFR and urine output, normalizing sodium, aldosterone, and ANP levels, and attenuating cerebrovascular leakage effects that were reversed by co‐administration with the ANP antagonist anantin, confirming an ANP‐mediated mechanism. HETA also improved brain antioxidant status, likely via its gallic acid, catechin, and ascorbic acid content, supporting *T. arjuna* as a multi‐organ, antioxidant‐rich candidate for nutraceuticals targeting hypoxia‐associated renal and cerebrovascular stress (Table 3) (Kumar et al. 2016). In male Balb/c mice, alcoholic TAE (25–100 mg/kg) reduced picrotoxin‐induced anxiety in a dose‐dependent manner, with effects comparable to diazepam. The extract also restored antioxidant defenses and modulated GABAergic, monoaminergic, and BDNF/CREB signaling, suggesting that its anxiolytic effects involve both redox and neurochemical regulation (Table 2) (Chandra Sekhar et al. 2017). Overall, the present evidence is largely confined to experimental toxin, ischemia, and hypoxia‐based animal models, and the specific roles as well as their translational relevance to human neurological disorders is yet to be established.

### 4.9 Diabetic Role

*T. arjuna* shows consistent anti‐diabetic activity across alloxan, STZ, and high‐fat diet models, and it works on both ends of the problem: insulin supply and glucose disposal. The bark extract (TAE) and its triterpenoid arjunic acid lower blood glucose and HbA1c, restore hepatic glycogen, and raise insulin/C‐peptide levels (Aamir et al. 2022; Biswas, Kar, et al. 2011; Manna, Das, et al. 2010; Manna and Sil 2012a; Tahsin et al. 2021). Arjunic acid/TAE treatment also upregulate IRS‐1 and PI3K/Akt signaling and boosts PGC‐1α (a key regulator of glucose uptake and mitochondrial function), largely by blocking JNK1/2‐driven suppression of IRS‐1 (Table 2) (Aamir et al. 2023; Fernandez‐Marcos and Auwerx 2011; Shengule et al. 2018). Besides this, *T. arjuna* is a direct modulator of the digestive and incretin‐related enzymes. It inhibits dipeptidyl peptidase‐4 (DPP‐IV) with an in vitro activity very close to sitagliptin/vildagliptin (Mohanty et al. 2019) and impairs α‐amylase and α‐glucosidase activities so as to reduce postprandial glucose spikes (Table 2) (Saha and Verma 2012). *In silico* docking studies show that phytochemicals of *T. arjuna*, such as arjunic acid, arjungenin, rutin, quercetin, gallic acid, and ellagic acid, have a strong binding affinity with DPP‐IV and carbohydrate‐digestive enzymes, often surpassing standard inhibitors (Mohanty et al. 2019; Tahsin et al. 2021). In vitro assay also verifies that the TAE is a very potent α‐amylase inhibitor (Saha and Verma 2012). Collectively, these data pinpoint that *T. arjuna* is capable of anti‐diabetic effects by the mechanisms of improved insulin action, enhanced glucose disposal, and inhibition of key enzymes involved in hyperglycemia.

Beyond glucose control, it protects the organs that diabetes typically damages. Hyperglycemia drives oxidative stress, antioxidant depletion, and apoptosis across the pancreas, liver, kidney, spleen, heart, and retina. Arjunic acid reverses these changes via interaction of its primary/secondary hydroxyl groups and carboxylic hydrogen impeding intrinsic (mitochondrial) apoptosis by stabilizing membrane potential, normalizes Bcl‐2 proteins, and blocks the caspase cascade, while also limiting PARP overactivation and the resulting NAD^+^/ATP depletion (Table 3) (Manna, Das, et al. 2010; Manna et al. 2009a, 2009b; Manna, Ghosh, et al. 2010; Manna and Sil 2012a, 2012b; Zhang et al. 2022). It also lowers the oxidative stress activated inflammatory signaling by impeding NF‐κB, p65, IκBα phosphorylation, and MAPKs (extracellular signal‐regulated kinase 1/2 (ERK1/2), p38, JNK) in numerous organs (Manna et al. 2009a, 2009b; Manna, Ghosh, et al. 2010; Manna and Sil 2012a, 2012b). Arjunic acid suppresses the inflammatory side of diabetic injury by NF‐κB/IκBα and MAPK (ERK1/2, p38, JNK) signaling and the TLR‐4/MyD88/NF‐κB pathway (Aamir et al. 2023), along with the polyol pathway implicated in diabetic kidney damage (Manna et al. 2009a; Manna and Sil 2012a) and restores splenic IL‐2/IFN‐γ and lowers TNF‐α reflecting better immune regulation and the prevention of immunosuppression caused by hyperglycemia (Manna, Ghosh, et al. 2010). In diabetic retina it stimulates AMP‐activated protein kinase (AMPK) – mammalian target of rapamycin (mTOR) – heme oxygenase‐1 (HO‐1) dependent autophagy, which in turn reduces oxidative damage and the release of inflammatory cytokines (Zhang et al. 2022). Functionally, this translates to real organ protection as TAE and arjunic acid ameliorate dyslipidemia to lower cholesterol, TGs, LDL/VLDLs and increase HDL (Khaliq, Parveen, Singh, Hussain, and Fahim 2013; Manna and Sil 2012b; Shengule et al. 2018), while reducing cardiac injury markers (CK, LDH, CK‐MB) (Manna and Sil 2012b; Mohanty et al. 2019). TAE and arjunic acid reduce TNF‐α, IL‐6, and IL‐1β (Aamir et al. 2023; Khaliq, Parveen, Singh, Gondal, et al. 2013; Khaliq, Parveen, Singh, Hussain, and Fahim 2013; Manna, Ghosh, et al. 2010), whereas cardiac vascular inflammation markers (VEGF, ICAM‐1, MCP‐1) (Manna and Sil 2012b). TAE preserves cardiovascular function like left ventricular performance, endothelin‐1, baroreflex sensitivity, autonomic neuropathy. Even in chronic models it doesn't lower blood glucose, indicating its cardioprotective effect isn't purely downstream of glycemic control but stands on its own antioxidant and anti‐inflammatory action (Khaliq, Parveen, Singh, Gondal, et al. 2013; Khaliq, Parveen, Singh, Hussain, and Fahim 2013). All together, these findings highlight *T. arjuna* as a strong antidiabetic agent by controlling blood glucose, supporting insulin signaling, and protecting organs from hyperglycemia‐mediated oxidative, inflammatory, as well as apoptotic injury (Table 3).

## 5 Translational Significance of *T. arjuna* on Clinical Patients

*T. arjuna* has demonstrated consistent cardioprotective effects in vitro and in vivo, and clinical studies report improved cardiac function, reduced angina frequency, and favorable immuno‐inflammatory and antioxidant modulation, supporting its potential as a translational phyto‐nutraceutical for cardiovascular disease management (Figure 4). Though, as discussed below, several of these studies present notable design limitations (Table 4).

**FIGURE 4:**
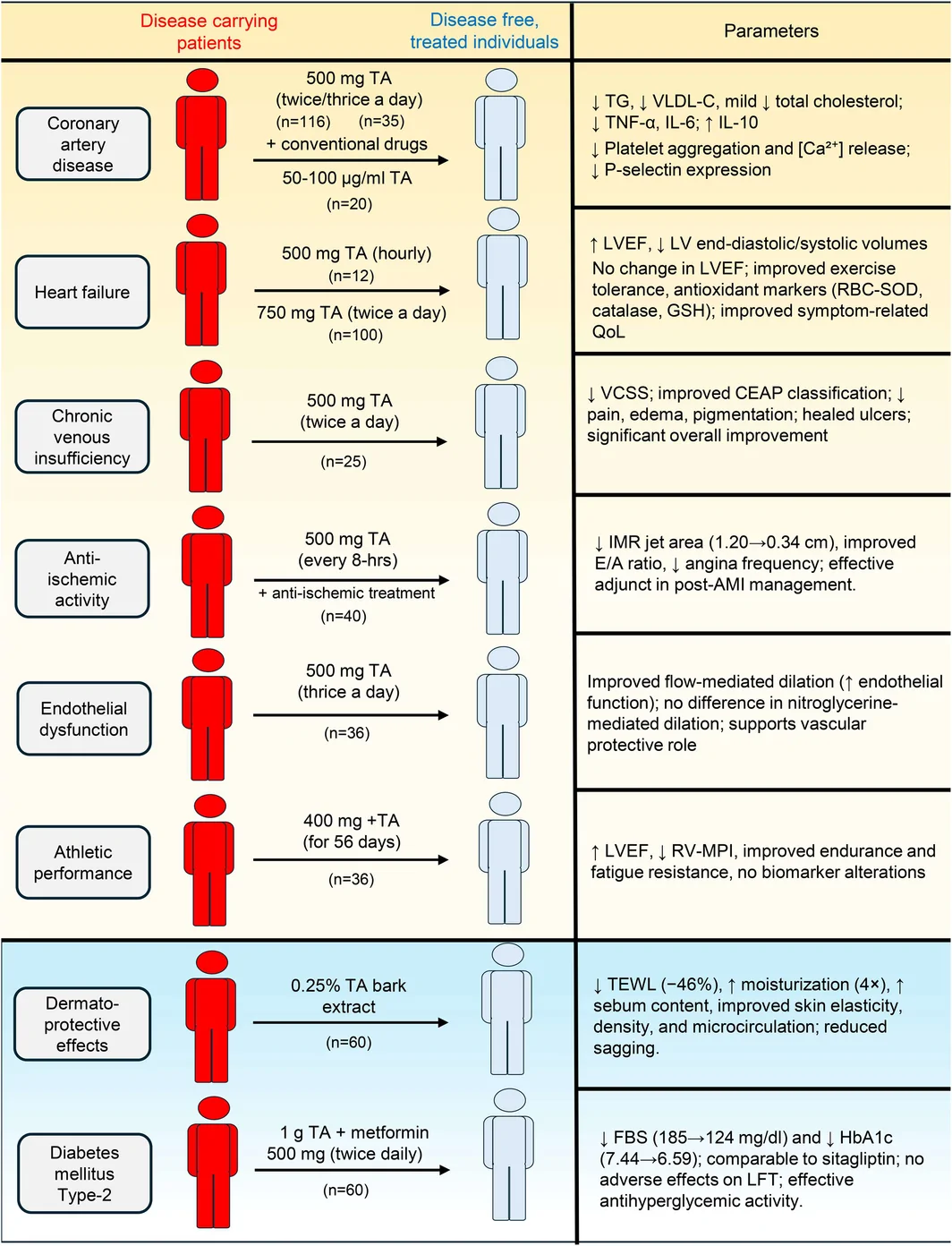
Summary of the clinical applications of *Terminalia arjuna* across different disease conditions: schematic representation of the therapeutic spectrum of *T. arjuna* in various human disorders. Clinical studies have demonstrated the significant efficacy of TA bark extract in coronary artery disease, heart failure, chronic venous insufficiency, diabetes mellitus type‐2, dermato‐protective effects, anti‐ischemic activity, and endothelial dysfunction. This figure summarizes the standardized dosages administered in the respective clinical trial, indicating *T. arjuna* as a multi‐target, safe, cardioprotective pharmaceutical agent.

**TABLE 4:** Clinical studies evaluating *Terminalia arjuna* across different diseases and conditions.

Type of disease/condition	No. of clinical patients	Type of study	Drug dose & application time	Clinical outcome	References
Coronary Artery Disease (CAD)	35 patients	Observational long‐term safety study	500 mg TAE thrice daily + conventional drugs; 4–5 years	No adverse effects on hematological, hepatic, or renal parameters; no hospitalization or mortality; safe for long‐term concurrent use.	Dwivedi et al. (2019)
	116 patients	Double‐blind clinical trial	500 mg TAE twice daily; 3–6 months	↓ TG, ↓ VLDL‐C, mild ↓ total cholesterol; anti‐inflammatory (↓ TNF‐α, IL‐6; ↑ IL‐10); well‐tolerated and safe; superior hypolipidemic activity vs. atorvastatin.	Kapoor et al. (2015)
	40 patients (20 control + 20 treated)	Controlled clinical study	50–100 μg/mL *Terminalia arjuna* for 3–5 min	Inhibited platelet aggregation and intracellular Ca^2+^ release; ↓ P‐selectin expression; exhibits anti‐thrombotic and anti‐atherosclerotic properties.	Malik et al. (2009)
Heart Failure	12 patients with refractory chronic CHF (Bharani et al. 1995)	Double‐blind, placebo‐controlled crossover study	500 mg TAE hourly for 2 weeks	↑ LVEF, ↓ LV end‐diastolic/systolic volumes, improved symptoms and NYHA class; benefits sustained for up to 28 months.	Bharani et al. (1995)
	100 patients	Double‐blind, randomized, placebo‐controlled	750 mg TAE twice daily for 12 weeks	No change in LVEF; improved exercise tolerance, antioxidant markers (RBC‐SOD, catalase, GSH); improved symptom‐related QoL.	Maulik et al. (2016)
Chronic Venous Insufficiency (CVI)	25 patients	Observational clinical study	500 mg TAE twice daily; assessed at 30 & 90 days	↓ Venous Clinical Severity Score (VCSS); improved CEAP classification; ↓ pain, edema, pigmentation; healed ulcers; significant overall improvement	Pratap Shankar and Ashwathykutty (2024)
Diabetes Mellitus (Type 2)	60 patients	Randomized comparative clinical trial	1 g *Terminalia arjuna* + metformin 500 mg twice daily; 3 months	↓ FBS (185 → 124 mg/dL) and ↓ HbA1c (7.44 → 6.59); comparable to sitagliptin; no adverse effects on LFT; effective antihyperglycemic activity.	Prakash et al. (2024)
Dermatoprotective Effects	30 + 30 postmenopausal women	Clinical skin efficacy study	0.25% TAE bark extract; 4–12 weeks	↓ TEWL (−46%), ↑ moisturization (4×), ↑ sebum content, improved skin elasticity, density, and microcirculation; reduced sagging.	Farwick et al. (2013)
Anti‐Ischemic Activity (Ischemic Mitral Regurgitation after AMI)	40 patients	Controlled comparative clinical study	500 mg *Terminalia arjuna* capsules 8‐hourly for 3 months	↓ IMR jet area (1.20 → 0.34 cm), improved E/A ratio, ↓ angina frequency; effective adjunct in post‐AMI management.	Dwivedi et al. (2005)
Athletic Performance/Ergogenic Study	32 exercising adults	Randomized, double‐blind, placebo‐controlled, parallel study	400 mg TAE (E‐OJ‐01) daily for 56 days	↑ LVEF, ↓ RV‐MPI, improved endurance and fatigue resistance, no biomarker alterations; safe and effective natural ergogenic aid.	Girandola and Srivastava (2017)
Endothelial Dysfunction (Smokers)	18 chronic smokers +18 non‐smoker controls	Double‐blind, placebo‐controlled, crossover study	500 mg TAE thrice daily for 2 weeks	Improved flow‐mediated dilation (↑ endothelial function); no difference in nitroglycerine‐mediated dilation; supports vascular protective role.	Bharani et al. (2004)

### 5.1 Coronary Artery Disease (CAD)

In a double‐blind, placebo controlled clinical trial, 116 CAD patients were given TAE (500 mg twice daily) for 3–6 months. The extract significantly reduced immuno‐inflammatory markers compared with placebo, lowered serum triglycerides (TG) and very‐low‐density‐lipoprotein cholesterol (VLDL‐C) more effectively than atorvastatin alone, along with inducing a modest reduction in total cholesterol. Transcriptomic, qPCR, and macrophage studies indicate that *T. arjuna* suppresses pro‐inflammatory cytokines, elevates IL‐10, and downregulates inflammatory pathways (Kapoor et al. 2015). These findings reflect changes in biochemical and inflammatory markers rather than hard clinical endpoints such as cardiovascular events. By contrast, a long‐term observational study (2011–2015) in 35 patients with chronic CAD and persistent angina or ischemic cardiomyopathy evaluated TAE (500 mg three times daily) alongside standard therapy (aspirin, beta‐blocker, ACE inhibitor, and statins). Treatment was well‐tolerated over 4–5 years, with no adverse effects on hematological, renal, or hepatic parameters. No patient required hospitalization, angioplasty, or bypass grafting, and there was no mortality in either the control or treated groups (S. Dwivedi et al. 2019). However, as an observational study with no randomized comparison group, it cannot distinguish *T. arjuna*'s effect from that of the standard cardiac therapy patients were already receiving. Another clinical study in CAD patients (20 per group) evaluated the anti‐platelet effects of TAE ex vivo. TAE significantly inhibited ADP‐induced platelet aggregation and reduced intracellular Ca^2+^ release and P‐selectin expression on platelets in a dose dependent manner. By attenuating Ca^2+^‐driven platelet activation and P‐selectin‐mediated leukocyte–platelet–endothelial adhesion, TAE acts as an anti‐platelet and anti‐thrombotic agent (Malik et al. 2009). As an ex vivo assay the results describe TAE's effect on isolated platelets and do not directly demonstrate a reduction in thrombotic events in patients.

### 5.2 Heart Failure

Two randomized, double‐blind, placebo‐controlled trials have evaluated *T. arjuna* in heart failure, with very divergent results. The smaller and earlier trial (*n* = 12, NYHA Class IV) subjected to TAE (500 mg hourly) and placebo for 2 weeks reported significant improvements in ventricular volumes and ejection fraction (35.33% ± 7.85% vs. 30.24% ± 7.13%; *p* < 0.005), with continued benefit over 20–28 months of follow‐up (Bharani et al. 1995). A considerably larger trial (*n* = 100, NYHA Class II) subjected to 750 mg TAE or placebo twice daily for 12 weeks did not replicate this ejection‐fraction benefit (24.3% ± 7.1% vs. 25.5% ± 7.7%; *p* = 0.4); instead, its benefits emerged only in secondary and exploratory measures—symptom and stability domains of the Kansas City Cardiomyopathy Questionnaire (KCCQ), 6‐min walk‐test distance (6‐MWT), and red‐blood‐cell antioxidant markers (Maulik et al. 2016). As with the ex vivo platelet findings discussed in Section 4, this in vitro result is a promising pharmacological signal but does not establish clinical efficacy or safety comparable to aspirin, whose safety profile is supported by decades of clinical use that AA has not yet undergone. These two trials illustrate a common pattern: the smallest study reports the largest effect on a hard functional measure, while the larger, better‐powered trial shifts the positive finding toward softer, secondary endpoints. This suggests the smaller trial's ejection‐fraction result should be treated as preliminary rather than established, since it may not hold up in a larger sample.

### 5.3 Chronic Venous Insufficiency (CVI)

Evidence for CVI comes from an observational study (*n* = 25) receiving TAE (500 mg twice daily) which reported significant improvement in venous clinical severity score from baseline to Day 90, alongside reduced pain, edema, inflammation, pigmentation, and improved ulcer healing. These findings highlight its potential as a vascular‐protective and dermatoprotective nutraceutical for CVI management, though future clinical trials will need to include a control/comparison group to confirm its benefit (Pratap Shankar and Ashwathykutty 2024).

### 5.4 Diabetes

A single trial (*n* = 60) compared *T. arjuna* plus metformin against sitagliptin plus metformin over 3 months, finding comparable reductions in fasting blood sugar and HbA1c between the two arms, with stable liver‐function parameters throughout. *T. arjuna* was an effective antihyperglycemic adjunct in diabetes management; however, without a placebo‐only arm, the trial cannot isolate *T. arjuna*'s independent contribution from that of metformin (Prakash et al. 2024).

### 5.5 Dermatoprotective Effects

The efficacy of TAE in protecting transepidermal water loss (TEWL), skin hydration, and elasticity was evaluated in 30 subjects treated with 0.25% TAE cream versus a vehicle control, and in another 30 postmenopausal women assessed for sebum content, cutaneous microcirculation, and skin density. Daily application of 0.25% TAE for 4 weeks reduced TEWL by 46% and increased skin moisturization fourfold compared with control, while also decreasing scaliness and improving elasticity. In postmenopausal women, 8–12 weeks of TAE use raised sebum content by 24%–62%, enhanced cutaneous microcirculation, improved skin thickness, and reduced sagging. These results demonstrate how *T. arjuna* exerts significant dermatoprotective effects, reinforcing its potential as a natural, nutraceutical‐grade ingredient for skin‐barrier support, hydration, and anti‐aging formulations (Farwick et al. 2013).

### 5.6 Anti‐Ischemic Activity

A short study reported a randomized trial (*n* = 40) evaluating whether *T. arjuna* could slow ischemic mitral regurgitation (IMR) progression after acute myocardial infarction, comparing standard anti‐ischemic therapy plus placebo against the same therapy plus *T. arjuna* (500 mg every 8 h). At 3 months, the *T. arjuna* group showed a significant reduction in IMR jet area (1.20 to 0.34 cm) and improved diastolic function, with reduced angina and only mild gastritis reported as a side effect (Dwivedi et al. 2005).

### 5.7 Athletic Performance

A separate randomized, double‐blind, placebo‐controlled, parallel‐group trial with 32 young exercising adults received 400 mg TAE (E‐OJ‐01) or placebo daily for 56 days during endurance training. The treatment significantly improved left ventricular ejection fraction (LVEF) and reduced right ventricular myocardial performance index (RV‐MPI), indicating enhanced cardiac performance along with improved endurance, lower perceived exertion, and a subjective sense of easier exercise, reflecting greater cardiac efficiency and fatigue resistance. These findings support E‐OJ‐01 as a safe, effective natural sports supplement with cardioprotective and ergogenic potential (Girandola and Srivastava 2017).

### 5.8 Endothelial Dysfunction

Bharani et al. (2004) conducted a double‐blind, placebo‐controlled, crossover study where 18 healthy male chronic smokers and age‐matched non‐smokers were randomly assigned to receive either TAE at a dose of 500 mg thrice a day or matching placebo for 2 weeks. Significant impairment in the brachial artery flow‐mediated dilation was observed in the extract treated chronic smokers than the healthy controls after 2 weeks, although the nitroglycerine‐mediated dilation rendered similar results in both the groups (Bharani et al. 2004).

Across these studies, *T. arjuna* shows a consistent pattern of benefit for cardiovascular and metabolic health, spanning improved heart function, reduced angina, and better blood sugar control. This clinical data represents an important step in bringing *T. arjuna's* traditional reputation into the realm of modern clinical science. At the same time, most of these trials were small, involved a single center, and only a few used a placebo‐controlled design; several instead relied on observational or comparison‐only designs that cannot fully separate *T. arjuna's* own effect from other factors. Larger, multi‐center, placebo‐controlled trials with clearly defined clinical endpoints would help confirm these promising early findings and establish *T. arjuna's* role in patient care.

## 6 Food Science‐Based Applications of *T. arjuna*

The diverse phytochemical composition of *T. arjuna* provides a foundation for its incorporation into functional foods, however their application in food‐science cannot be inferred from pharmacological activity alone, factors like solubility, interactions with the food matrix, processing stability and gastrointestinal bioaccessibility all influence its effective delivery (Vareltzis et al. 2026). In a dairy experiment on hypercholesterolaemic Wistar rats, microencapsulated TAE was blended into a vanilla chocolate milk drink and evaluated in a 60‐day feeding trial, where it produced significant reductions in triglycerides, total cholesterol, LDL‐ and VLDL‐cholesterol, and atherogenic index relative to the high‐cholesterol control. The formulation of the fortified beverage was optimized to maintain the products flavors, physicochemical and sensory properties while also retaining the bioactive potential of *T. arjuna*. This finding provides a basis for further investigation of such beverages in human trials as a potential dietary strategy (Sawale et al. 2016). In another study, *T. arjuna* bark extract was incorporated into clarified butterfat (ghee) as a natural antioxidant, improving radical‐scavenging activity and oxidative stability. Accelerated oxidation testing showed that 7% w/w ethanolic bark extract extended the shelf life of ghee several‐fold compared with untreated ghee (Jain et al. 2026). In the meat‐emulsion study, ethanolic TAE was added directly to chevon (goat) meat batter at four graded concentrations alongside a positive synthetic‐antioxidant control (BHA). The results showed concentration‐dependent antioxidant activity, with the higher concentration significantly reducing TBARS, peroxide value and free fatty acids during a 9‐day storage period. The treated meat batter also showed significantly lower spoilage microorganisms, including coliforms, psychrophilic bacteria and yeast/molds (Singh et al. 2026). In *Labeo rohita* , dietary incorporation of *T. arjuna* bark powder has also been investigated as a food/feed‐based delivery approach. In the study, supplementation of bark powder at 10 g/kg feed for 90 days enhanced hematological and immune parameters (Meena et al. 2024). A separate study reported dose dependent changes in the expression of interferon‐associated immune markers including STAT1, ISG15 and Mx, with 10 g/kg producing the strongest response for Mx. These findings highlight that optimizing the inclusion level of *T. arjuna* when delivered through a conventional dietary matrix (Meena et al. 2022). Beyond its direct incorporation into foods, *T. arjuna* gum has shown useful properties as a natural biopolymer for controlled delivery. Gum‐based microbeads provided high drug‐entrapment efficiency and pH‐responsive release, while gum/alginate gels demonstrated prolonged release under physiological conditions, suggesting that the functional properties of *T. arjuna* gum could be explored further for food‐grade encapsulation and delivery of its bioactives (Basharat et al. 2025; Noreen et al. 2020). The medicinal plant was also incorporated into calcium‐alginate edible films for chevon sausages, where 0.5% and 1.0% formulations improved oxidative and microbial stability during refrigerated storage. This highlights the use of it as an active packaging ingredient for maintaining food quality and extending shelf life (Kalem et al. 2018). Overall, these studies show that *T. arjuna* can be incorporated into foods and active packaging, and its effectiveness is influenced by different formulation, dose and storage conditions.

## 7 Safety, Toxicology, Pharmacokinetics and Herb‐Drug Interactions

Though there has been substantial research on the medicinal uses of *T. arjuna*, there still remains uncertainty about its safety and potential side effects. The data available is quite diverse in terms of various body parts used, doses, preparations, and more.

### 7.1 Preclinical Toxicological Evidence

In terms of preclinical studies, short‐term systemic toxicity appears low in various preparations of *T. arjuna*, yet the results differ depending on the specific preparation and the endpoint. No mortality occurred after a single oral dose of 2000 mg/kg of HETA; when administered daily at 100–1000 mg/kg in rats for 28 days, there were no treatment‐related hematological, biochemical, urinalysis and organ weight changes. Thus, 1000 mg/kg/day was found to be the highest dose that produced no observable harmful effects (Thomas and Ethiraj 2023). A similar conclusion can be drawn based on a methanolic bark extract, which was not lethal at a dose of 2000 mg/kg and showed no systemic toxicity after 28 days administration at 1000 mg/kg/day in male mice (Suganthy et al. 2018). In contrast, a methanolic leaf extract had a LD50 of 900 mg/kg, but 200 mg/kg/day for 28 days caused no hematological, biochemical, and histological changes (Biswas, Karan, et al. 2011). Thus, safety data from one extract or plant part cannot be directly extrapolated to another.

Recent data also shows that *T. arjuna* extracts do not cause notable genetic damage. Hydroalcoholic bark extract was negative in the Ames test and did not cause significant structural and numerical chromosome aberrations in Chinese hamster ovary cells (Thomas and Ethiraj 2023). A similar conclusion can be made about methanolic bark extract. It was found not to be mutagenic in strains TA98 and TA100 of *Salmonella typhimurium* and showed no significant DNA damage in the comet assay as well as cytotoxic/hemolytic activity (Suganthy et al. 2018). However, these results pertain to selected preparations only and cannot be considered as a replacement for long‐term in vivo genotoxicity/carcinogenicity experiments. Most importantly, the research has established the effects that are not observed using standard toxicity tests. Thus, it has been found that in male rats, the alcoholic bark extract of *T. arjuna* given at 150 mg/kg/day resulted in a reduction of testicular steroidogenic enzyme activity, testosterone level and spermatogenesis in 21 days without significant changes at 7 and 14 days (Chattopadhyay et al. 2014). The other study indicated that bark extract caused thyroid hormone decrease and elevation of hepatic lipid peroxidation in euthyroid rats (Parmar et al. 2006). Importantly, thyroid suppression in thyroxine‐fed animals correlated with decreased oxidative stress in heart tissue indicating that beneficial effect in pathology could be harmful for normal physiology. These facts do not oppose the first and second study and cannot eliminate the possibility that regular biochemical and histological testing can miss certain endocrine and reproductive effects.

To conclude, TAE possesses relatively good short‐term systemic and no significant genotoxicity; however, the information about chronic, reproductive, endocrine, and developmental safety is lacking.

### 7.2 Pharmacokinetics and Potential Herb‐Drug Interactions

Data on pharmacokinetics and herb‐drug interactions are mostly preclinical. Both aqueous and alcoholic bark extracts inhibited cytochrome P450 (CYP) 3A4, CYP2D6, and CYP2C9, but arjunic acid, arjunetin, and arjungenin demonstrated weak inhibitory activity in human liver microsomes (Varghese et al. 2015). Aqueous, hydroalcoholic, and alcoholic extracts inhibited CYP1A in rat and human microsomes, but individual arjunic acid and arjungenin were inactive (Varghese et al. 2014). Bioactivity‐guided fractionation showed that CYP3A and CYP2D inhibition was confined to certain fractions of a methanolic bark extract (Varghese et al. 2018). Thus, all these studies suggest that interaction risk is more associated with the composition of the whole extract than with individual triterpenoid biomarkers.

Microsomal inhibition is not a reliable indicator of in vivo interaction risk. Despite inhibition of CYP1A in vitro, administration for 1 week of aqueous bark extract did not have any effect on phenacetin pharmacokinetics in rats (Varghese et al. 2014). Conversely, CYP2D inhibition was linked to changes in metoprolol pharmacokinetics and pharmacodynamics in rats (Varghese et al. 2016). Importantly, area under the concentration‐time curve and maximum plasma concentration of metoprolol were decreased in spite of the CYP2D inhibition. All this means that interaction risk can vary with the enzyme, substrate and preparation. The choice of formulation also affects systemic availability. Higher maximum plasma concentration and area under the concentration‐time curve were achieved in the case of a bark‐extract nanosuspension compared to a coarse suspension, which was translated to a 1.33‐fold relative increase in oral bioavailability according to quercetin equivalents (Zafar et al. 2020). This finding makes extrapolations even more problematic between various *T. arjuna* products. Potential drug interactions might also have a pharmacodynamic nature. Bark ethanol extract inhibited platelet aggregation, intracellular calcium mobilization, and P‐selectin expression in vitro in platelets of both patients suffering from coronary artery disease and healthy volunteers (Malik et al. 2009). Such an effect suggests possible additive action of the extract in combination with antiplatelet and anticoagulant drugs. Yet, no bleeding outcomes or experiments with combinations of drugs were performed. Similar lack of clinical consequences was found for the long‐term co‐administration with aspirin, beta blockers, angiotensin‐converting enzyme inhibitors, and statins (S. Dwivedi et al. 2019) in a small sample, but it doesn't exclude the possible pharmacokinetic or pharmacodynamic drug interactions.

Thus, *T. arjuna* has potential drug interaction risks, although their clinical significance is unknown. There is a need for controlled human pharmacokinetic and drug interaction studies using standardized formulations of *T. arjuna*.

## 8 Future Perspectives

Moving forward, for *T. arjuna* to emerge as an integral component of the next generation of phytomedicine and nutraceuticals, the current trend of isolated investigations on efficacy would need to be replaced by larger clinical trials which utilize a standardized, chemical analysis‐based formulation of the bark extract, in combination with optimized dosages and prolonged effects. Future prospective investigations in different areas—such as cardiovascular disorders, metabolism and the gut microbiome employing harmonized endpoints to identify the underlying mechanisms of action. In parallel, innovative approaches of formulations through nanotechnology or micro‐encapsulation; novel matrix systems to create functional foods; and advanced synergetic synbiotic systems are potential paths to enhanced bioavailability, targeted delivery, and sustained patient compliance. Multiomic considerations in the design of such nutraceuticals may allow for precision nutrition, matching specific phenotypes with *Terminalia* formulations. Along with this, responsible agriculture practices, quality control processes, and monitoring of adverse events will propel *T. arjuna* into a new era as a validated functional ingredient within the emerging field of the nexus between food and health.

## 9 Discussion

Long before its pharmaceutical properties were characterized, *T. arjuna* entered the human diet in the form of a cardiotonic, bark powders, capsules, decoctions and fermented tonics. A hydro‐alcoholic formulation called “Arjunarishta” made with *T. arjuna* aids as an excellent treatment for gastrointestinal diseases (Gairola et al. 2013; Kapoor et al. 2014). Arjunarishta enhanced antioxidants such as SOD, CAT, and GSH, decreased oxidative stress parameters like NO, MDA, and MPO, and inhibited pro‐inflammatory cytokine expression such as TNF‐α, IL‐1β, and IL‐6 in a TNBS‐induced colitis mouse model (Cota et al. 2020). In the aquaculture industry, *T. arjuna* bark powder is widely used as a nutraceutical which functions as an environmentally friendly feed additive with the potential to improve fish health, growth and survival (Meena et al. 2025). TAE and bioactive compounds like AA, arjunic acid, and casuarinin demonstrate pleiotropic effects across oxidative stress, inflammation, apoptosis, and metabolic dysregulation in preclinical models. These actions involve key pathways such as p53/Fas–FasL mediated cell cycle regulation (Kuo, Hsu, Lin, Chang, and Lin 2005), JNK/CHOP associated ER stress (Joo et al. 2016), and PPAR/TGF‐β‐TAK1 signaling (Bhat et al. 2018), often accompanied by restoration of redox balance via SOD, CAT, GPx upregulation and GSH preservation (Verma and Vinayak 2009).

AA exemplifies multifunctionality by inhibiting TGF‐βR1 in EAC to curb tumor progression (Elsherbiny and Al‐Gayyar 2016) and PPAR associated pathways (Bansal et al. 2017), However, these findings are predominantly preclinical and may vary according to the extract, constituent and experimental model. Nanotechnology based formulations like self‐assembled arjunolic vesicles (Manna et al. 2020) or TA‐AgNPs/SeNPs amplify selective cytotoxicity in MCF‐7/HeLa/PC3 cells via ROS/TNF‐α overload (Prasannaraj et al. 2016), but these are in an early translational stage with limited pharmacokinetic and toxicity studies.

Clinically, the evidence is much more limited than the extensive preclinical evidence. Cardiovascular studies provide the strongest human evidence. TAE improves endothelial function in smokers (Bharani et al. 2004), reduces angina/IMR post MI (Dwivedi et al. 2005), enhances NYHA II/III heart failure, lowers HbA1c/FBS in T2DM comparable to sitagliptin (Prakash et al. 2024), and alleviates chronic venous insufficiency (Pratap Shankar and Ashwathykutty 2024). Even with strong preclinical evidence, clinical trials are hindered by poor sample sizes, non‐standardized preparations of extracts, and poor design with bad randomization and lack of blinding.

The safety evidence is relatively reassuring but it is insufficient for establishing long‐term safety across all preparations. Because toxicological findings vary with plant part, extraction method and dose, and therefore safety observed for one preparation cannot be generalized to another (Thomas and Radhakrishnan 2023; Biswas, Karan, et al. 2011; Chattopadhyay et al. 2014). From a food‐science perspective, current studies demonstrate the feasibility of incorporating *T. arjuna* into food matrices such as dairy beverages and ghee, while encapsulation may improve stability and delivery (Sawale et al. 2016; Jain et al. 2026; Vareltzis et al. 2026). With better long term food safety data, human bioavailability and better bioaccessibility, it can become a valuable food ingredient.

Taken together, *T. arjuna* can be regarded as part of a holistic approach to health management, as an adjunct to conventional nutrition and medication, showing promising therapeutic potential.

## Author Contributions

**Ishani Banerjee:** writing – original draft, methodology, writing – review and editing. **Fahika Nazeerulla:** writing – original draft, methodology, writing – review and editing. **Manash Kumar Paul:** validation. **Sujit Kumar Bhutia:** validation, writing – review and editing. **Deblina Basu:** writing – review and editing. **Subhadip Mukhopadhyay:** conceptualization, writing – original draft, writing – review and editing, methodology, investigation, validation, supervision.

## Funding

This work is supported by research facilities provided through Manipal School of Life Sciences, Manipal Academy of Higher Education (MAHE), Manipal, India. SM, MKP acknowledge the intramural and MRB funding, respectively, from MAHE, Manipal, India. SM acknowledges the support from Anusandhan National Research Foundation (ANRF), Government of India, through ANRF‐ARG grant (ANRF/ARG/2025/005742/LS). The authors gratefully acknowledge infrastructure funding from DST‐FIST, K‐FIST, TIFAC‐CORE, DBT‐BUILDER, VGST Karnataka, Manipal School of Life Sciences and Manipal Academy of Higher Education, Manipal, Karnataka, India. MKP gratefully acknowledge the financial support received from the Indian Council of Medical Research through the Intermediate Research Grant (IIRPIG‐2025‐01‐01249) and Small Grant (IIRPSG‐2025‐01‐01645). He also acknowledges funding provided by Vision Group on Science and Technology under the K‐FIST L2 scheme (K‐FIST L2/GRD No: 1205/2023‐2024), and the Department of Biotechnology (BT/PR65/207/MED/30/2586/2025). SM and MKP also acknowledge the Centre for Organoid Bioengineering, Bioprinting, Biobanking, and Biomedical Sciences (COBS), MAHE, Manipal.

## Conflicts of Interest

The authors declare no conflicts of interest.

## Data Availability

The authors have nothing to report.
